# Artificial intelligence assisted common maternal fetal planes prediction from ultrasound images based on information fusion of customized convolutional neural networks

**DOI:** 10.3389/fmed.2024.1486995

**Published:** 2024-10-29

**Authors:** Fatima Rauf, Muhammad Attique Khan, Hussain M. Albarakati, Kiran Jabeen, Shrooq Alsenan, Ameer Hamza, Sokea Teng, Yunyoung Nam

**Affiliations:** ^1^Department of Computer Science, HITEC University, Taxila, Pakistan; ^2^Department of Artificial Intelligence, College of Computer Engineering and Science, Prince Mohammad Bin Fahd University, Al Khobar, Saudi Arabia; ^3^Computer and Network Engineering Department, College of Computing, Umm Al-Qura University, Makkah, Saudi Arabia; ^4^Department of Information Systems, College of Computer and Information Sciences, Princess Nourah Bint Abdulrahman University, Riyadh, Saudi Arabia; ^5^Department of ICT Convergence, Soonchunhyang University, Asan, Republic of Korea

**Keywords:** ultrasound, maternal fetal, residual blocks, deep learning, information fusion, optimization

## Abstract

Ultrasound imaging is frequently employed to aid with fetal development. It benefits from being real-time, inexpensive, non-intrusive, and simple. Artificial intelligence is becoming increasingly significant in medical imaging and can assist in resolving many problems related to the classification of fetal organs. Processing fetal ultrasound (US) images increasingly uses deep learning (DL) techniques. This paper aims to assess the development of existing DL classification systems for use in a real maternal-fetal healthcare setting. This experimental process has employed two publicly available datasets, such as FPSU23 Dataset and Fetal Imaging. Two novel deep learning architectures have been designed in the proposed architecture based on 3-residual and 4-residual blocks with different convolutional filter sizes. The hyperparameters of the proposed architectures were initialized through Bayesian Optimization. Following the training process, deep features were extracted from the average pooling layers of both models. In a subsequent step, the features from both models were optimized using an improved version of the Generalized Normal Distribution Optimizer (GNDO). Finally, neural networks are used to classify the fused optimized features of both models, which were first combined using a new fusion technique. The best classification scores, 98.5 and 88.6% accuracy, were obtained after multiple steps of analysis. Additionally, a comparison with existing state-of-the-art methods revealed a notable improvement in the suggested architecture’s accuracy.

## 1 Introduction

Preterm birth (PTB) is a challenging obstetrical syndrome that significantly contributes to newborn health problems and mortality (1). The term “maternal-fetal” pertains to the bond between a pregnant mother and her unborn baby, encompassing their health and interactions (2). Detecting fetal abnormalities increases the likelihood of successful treatment (3). During the first trimester of pregnancy, the risk of miscarriage is higher than in later trimesters (4). It is a critical period for fetal development, and various factors can contribute to this increased risk. On a global scale, approximately 15 million infants are born preterm each year, constituting over 10% of all deliveries worldwide. Preterm birth remains a significant public health concern due to its potential impact on neonatal health and mortality (5). In Brazil, the Ministry of Health currently recommends a minimum of six prenatal visits throughout pregnancy—one during the first trimester, two during the second, and three during the third. Nevertheless, if there are any gestational complications, this number should be increase (6). Approximately 2 million premature births were reported by the World Health Organization in 2019. The majority of these might have been prevented by ensuring safe, high-quality care, implementing timely emergency interventions, and keeping accurate records (7). Early obstetric ultrasound determines the location of the pregnancy, detects fetal cardiac activity, estimates gestation, identifies multiple pregnancies and fetal anomalies, reduces the likelihood of inductions after term, and enhances the experience of pregnancy (8). Vietnam’s Ministry of Health recommends at least four antenatal care (ANC) visits. In urban and rural areas, nearly all women attend at least one. As of the early 21st century, ANC includes three routine ultrasounds and a third-trimester growth scan (9). During a cross-sectional investigation conducted in Poland, the importance of physical activity before and during pregnancy was highlighted, emphasizing its role in reducing adverse perinatal outcomes (10). A healthy pregnancy not only promotes long-term maternal well-being but also positively impacts the child’s development from infancy to adulthood (11). Maternal health is strongly correlated with a reduced risk of chronic conditions in children, such as obesity, diabetes, and cardiovascular disease (12). Despite improvements in maternal health outcomes across Europe, significant disparities persist. Some countries report maternal mortality rates up to four times higher than the European Union average. For instance, in 2021, the EU average was 4 deaths per 100,000 live births, while Romania and Bulgaria reported around 16 per 100,000 (13). Early and continuous prenatal care can reduce maternal mortality and morbidity by up to 50% and improve birth outcomes by 60%, according to WHO estimates (14).

According to a survey conducted in the United States, infant mortality rates dropped by 14% from 2007 to 2017. However, in some developing countries, a lack of publicly available resources to address such health conditions has serious consequences for many infants and pregnant women (15). Medical imaging technology has improved significantly in recent years, making fetal imaging a more mature discipline (16). Ultrasound (US) (17) is frequently employed in clinical practice due to its ability to provide real-time imaging, low cost, and lack of radiation. During the ultrasound examination, various important aspects of the fetus must be identified, sized, grown, oriented, and gestational age determined (18). The first trimester of pregnancy is also an excellent time to use ultrasonography to identify any potential abnormalities in the uterus or cervix (19). Fetal movements and maternal respiration introduce motion artifacts that corrupt the data (16). Automated organ localization is challenging due to fetal positioning and placental location irregularities. Moreover, the complexity of texture differentiation increases due to the presence of both fetal and maternal tissues in ultrasound imaging (20). In cases of multiple pregnancies, data processing and analysis become even more complicated as fetal structures are duplicated (21). Such variability has the potential to result in misdiagnosis, such as inaccurately estimating fetal growth, or even worse, it can lead to missed conditions, making the process time-consuming (4). To guarantee accurate interpretation of data obtained from these examinations, the task is performed manually by trained research technicians and later validated by a senior specialist in maternal-fetal care (22).

However, the crucial task of determining the fetus’s orientation and assessing vital biometric measurements, such as abdominal circumference or femur length, necessary for determining gestational age, currently depends solely on the expertise of trained sonographers and physicians (18). Well-organized retrospective data is vital for studies on fetal growth and diseases (3). Therefore, implementing an automated system capable of performing this task would enhance cost-effectiveness and potentially reduce errors and mistakes (22). The demand for automatic computer-aided diagnosis (CAD) to assess the caliber images from ultrasound is increasing, mainly to assist junior doctors. This trend, known as “intelligent ultrasound,” is driven by the quick advancement of medical imaging techniques (23). To observe and detect certain anatomical structures using machine learning and neural networks, many dedicated research works have focused on the excellent evaluations of unborn offspring ultrasound pictures (24). Integrating deep neural networks (DNN) in CAD has proven highly advantageous, resulting in decreased errors and improved measurement efficiency compared to healthcare professionals and conventional CAD tools (18). Preprocessing is performed on the obtained 2D ultrasound pictures to enhance feature extraction and address issues related to redundant information. The feature extraction process involves analyzing objects and images to identify the distinctive characteristics representing different object classes (25). Feature Selection is performed to selecting relevant features and then in maternal-fetal medicine, different imaging modalities (e.g., ultrasound, magnetic resonance imaging) or various types of data (e.g., physiological measurements, biomarkers) could be fused to enhance the understanding and assessment of maternal and fetal health (26).

Artificial intelligence (AI) techniques have enabled recent breakthroughs in obstetrics and gynecology, allowing for rapid and automated identification and measurement of both normal and abnormal ultrasound findings (27). Despite this development, no research has been conducted to evaluate fetal occiput position during labor using an AI-based model (27). Deep Learning (DL) has made significant progress in image recognition, especially with Convolutional Neural Networks (CNNs), and artificial intelligence has grown substantially over the past decade. DL, particularly in medical imaging, is rising, showing promise in magnified visuals, heart disease diagnostics, and gynecological imaging (28). In fetal imaging, DL has become a valuable tool for educating and training young and inexperienced medical professionals, offering benefits in recognizing fetal development and measuring prenatal biometry (29). Its automation potential reduces variability and examination times, enhancing workflow efficiency and reducing long-term fatigue and injuries. DL algorithms excel at identifying fetal standard planes, including the brain, thorax, femur, heart, and abdomen (30).

This study aims to evaluate the potential of deep convolutional neural networks in automating the classification of typical maternal-fetal ultrasound planes. The ultimate aim is to enhance diagnostic accuracy and elevate clinical performance, presenting a promising future for healthcare. A new deep learning framework is proposed in this work for accurate classification of common maternal fetal classification using Ultrasound images. The major contributions of this work are as follows:

- We have introduced two novel convolutional neural network architectures, namely the 3-Residual and the 4-residual-block models, each designed to enhance efficiency compared to ResNet 18 and ResNet 50.
- Proposed 3-Residual model incorporates three residual blocks with fewer parameters than ResNet 18 and ResNet 50, ensuring computational efficiency while maintaining competitive performance.
- Proposed four-residual-block model introduces additional hidden layers and a limited number of max-pool layers, achieving efficiency through a reduced parameter count compared to ResNet 18 and ResNet 50.
- Our proposed models prioritize streamlined architectures without compromising accuracy, distinguishing them as efficient alternatives to the benchmark ResNet models.
- We have proposed an enhanced version of the Generalized Normal Distribution Optimization algorithm to eliminate irrelevant features from the extracted features. This modification aims to select the best features, ultimately enabling us to achieve the highest accuracy.

## 2 Literature review

A few deep-learning techniques have been introduced in the literature to classify common maternal fetuses from Ultrasound images. Selvathi and Chandralekha (31) employed a complex convolutional neural network method to identify regions to examine ROI in ultrasound images containing fetal biometrics and organ structures. The image feature was evaluated using AlexNet (32), GoogleNet (33), and CNN based on the depiction of key fetal biometric structures within the ROI. The presented work achieved 90.43, 88.70, and 81.25% accuracy with AlexNet, GoogleNet, and CNN, respectively, while classifying 400 images, including normal and abnormal ultrasound data. Nurmaini et al. (34) developed DL approaches for diagnosing CHDs in fetal ultrasound scans, with DenseNet201 as the top classifier for seven CHD categories and normal cases. All feature maps from previous layers are merged in the DenseNet architecture to facilitate information distribution. In contexts involving both intra-patients and other patients, DenseNet201 achieved excellent sensitivity, specificity, and accuracy.

The study aims to support front-line sonographers and enhance CHD diagnostics with expert fetal cardiologist assistance. Płotka et al. (35) presented a versatile deep-learning architecture called FUVAI, specifically developed for analyzing fetal ultrasound videos. FUVAI performs multiple tasks simultaneously within fetal ultrasound videos, including localizing standard planes, classifying and measuring unborn biometric credentials, and estimating maternal age and fetal dimensions from the video sequences. The results demonstrated that FUVAI achieved performance equivalent to that of humans, as agreed upon by experienced audiologists. According to Rahman et al. (19), the experiment aimed to employ Artificial Neural Networks (ANNs) explored in predicting obstetrical outcomes in samples of low-risk pregnant women. The study used an Artificial Neural Network (ANN) trained with eight input variables representing obstetrical history to predict preterm birth and high-risk preterm birth outcomes. Moreover, the researchers refined the model by excluding cases that involved free-flow oxygen resuscitation. The refined high-risk preterm delivery model achieved 54.8% sensitivity compared to the preterm birth model without artificial transportation. Utilizing the YOLOv4 (21) target detection algorithm, the method incorporated additional attention mechanisms to pinpoint crucial anatomical structures. The strategy yielded amazing results, attaining an impressive mean identification precision of 94.16% for six formations, a precision rate of 97.20% for the regular median sagittal plane, and an accuracy level of 99.07% for the typical retro-nasal triangle perspective. As a result, automatic acquisition in early pregnancy ultrasound screenings may become feasible with the developed method.

**Figure 1:**
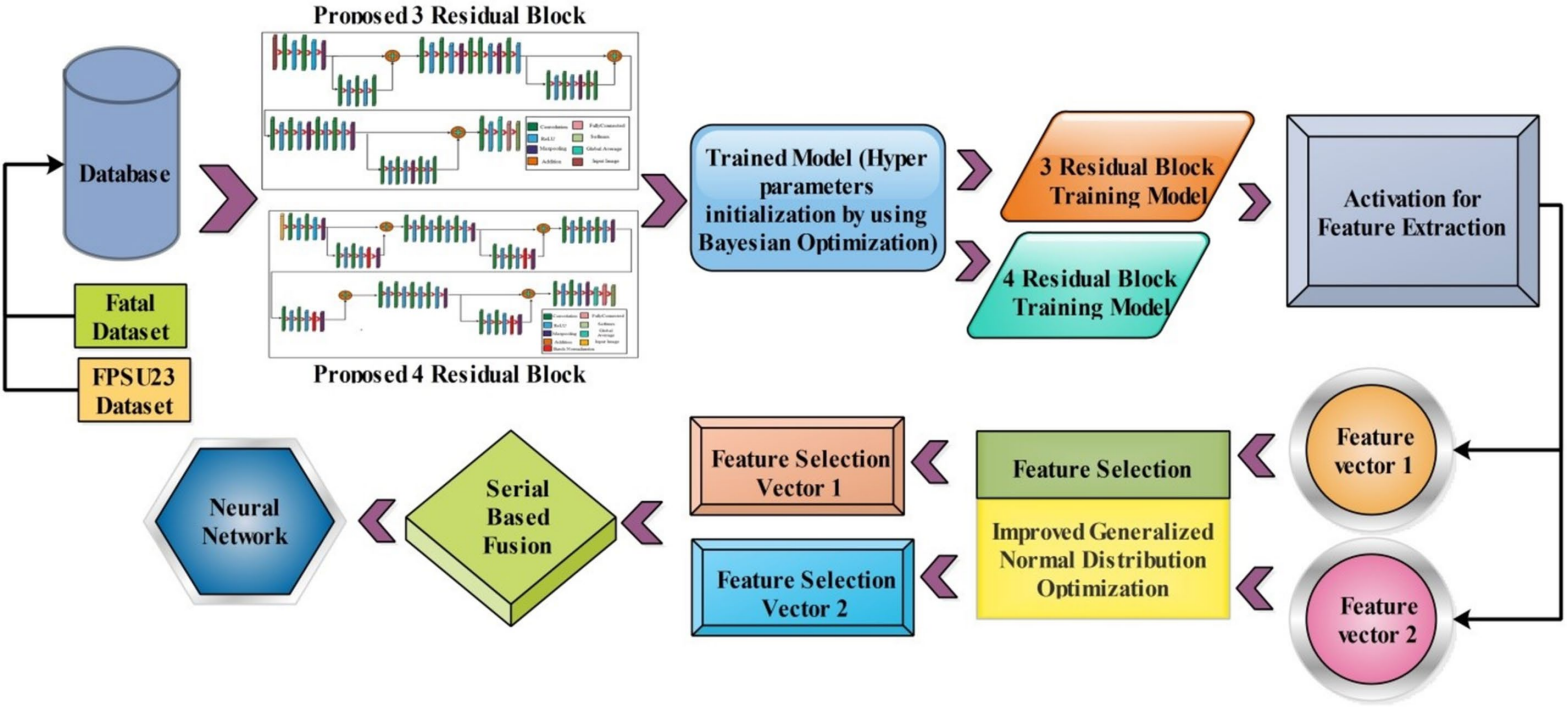
A framework for maternal-fetal classification using ultrasound images.

In 2022, Arroyo et al. (36) developed an automated diagnostic framework to improve obstetric care in under-resourced communities with limited ultrasound imaging. The framework utilized a standardized VSI protocol and the UNet deep learning algorithm, eliminating the need for experienced sonographers or radiologists. The UNet model showed remarkable accuracy in evaluating fetal presentation, placental location, and biometric measurements, potentially reducing healthcare disparities in underserved regions. Torrents-Barrena et al. (16) focus on efficient segmentation techniques for fetal MRI and 3D ultrasound images of intrauterine tissues. Radiomic features are used to characterize anatomies, and machine learning helps identify optimal features for accurate segmentation using support vector machines. Utilizing DeepLabV3+ or BiSeNet in MRI applications and employing PSPNet or Tiramisu for 3D ultrasound imaging and specific radiomic features further improves fetal and maternal tissue segmentation, advancing surgical planning and segmentation techniques. To enhance tissue characterization, Figure 1 shows the 10 top engineering features and network framework elements for each anatomy. According to a global survey, approximately 8% of the population is impacted by genetic syndromes, but genetic diagnoses are typically made after birth. To address this, Tang et al. (37) introduced a new fully automated prenatal screening algorithm called Pgds-ResNet, which was developed, utilizing deep neural networks to identify high-risk fetuses with different genetic diseases. The algorithm found that diagnostic information could be extracted from fetal features such as the nose, jaw, and forehead. It is essential to understand that this deep-learning-based tool is a diagnostic aid to doctors, streamlining the process without replacing their expertise. Mirzamoradi et al. (5) introduced an artificial neural network (ANN) approach to early predict preterm birth (PB), enabling physicians to initiate treatment sooner and reduce the risk of infant morbidity and mortality. The study employs a feed-forward ANN with seven hidden neurons for PB prediction and achieves an accuracy of 79.03% in classifying subjects into normal and PB categories. Li et al. (38) employed ResNet50, a pre-refined model based on deep multilayer neural networks, to reduce inter-observer variation in identifying fetal growth. Using the model, Crown Rump Length (CRL) images between 11 and 13 + 6 weeks were classified as either accurate or inaccurate. Through the implementation of a skip link strategy for constructing a more intricate network tuned to the particular tasks hyper parameters, the system achieved an 87% accuracy in identifying the images across preparation, validation, and test datasets from a real dataset containing 900 CRL images, with 450 images being correct and 450 being incorrect.

Prabakaran et al. (18) introduced FPUS23, a novel fetal phantom ultrasound dataset tailored to diagnose fetal biometric values, determine fetus orientation, recognize anatomical features, and outline bounding boxes for fetal phantom anatomies at 23 weeks gestation. It comprised 15,728 images employed to train four Deep Neural Network models using a ResNet34 backbone for feature detection. The assessment demonstrated that models trained on FPUS23 enhanced accuracy by 88% when applied to actual ultrasound fetus datasets. Włodarczyk et al. (39) developed a ConvNet for automated classification of prenatal ultrasound images and preterm birth detection. The CNN efficiently handled various cervix types in transvaginal ultrasound images, using image features to predict autonomous preterm birth. The authors evaluated three widely recognized network models to address the cervix segmentation challenge: U-Net, Fully Convolutional Network, and Deeplabv3. It achieved impressive results with high segmentation accuracy (with a mean Jaccard coefficient index of 0.923 ± 0.081) and exceptional classification responsiveness (0.677 ± 0.042), while maintaining a low false positive rate (3.49%). Baumgartner et al. (40) presented a convolutional neural network (CNN) technique to autonomously recognize and pinpoint 13 typical fetal perspectives within 2-D ultrasound data. Xie et al. (41) discovered 2,529 and 10,251 abnormal pregnancies, respectively, confirmed by ultrasounds, follow-ups, or autopsy. The training data focused on lesion location, skull segmentation, and classification of normal and pathological images.

Lim et al. (42) gathered a diverse dataset of 33,561 de-identified two-dimensional obstetrical ultrasound images collected between January 1, 2010, and June 1, 2020. The dataset was classified into 19 unique classes based on standard planes and split into training, validation, and testing sets using a 60:20:20 stratified technique. Using a convolutional neural network framework and transfer learning, the standard plane classification network achieved impressive results, with 99.4% accuracy and a 98.7% F1 score. Furthermore, the diagnostic usability network performed exceptionally well, achieving 80% accuracy and an F1 score of 82%.

Ferreira et al. (26) employed medical data from 808 participants and 2024 ultrasound pictures to create AI models that predict vaginal delivery (VD) and cesarean birth (CS) results following induction of labor (IOL). The best model, based solely on clinical data, achieved an F1-score of 0.736 and a PPV of 0.734. Ultrasound-based models, particularly those using femur images, showed lower accuracy. An ensemble model combining clinical data with femur images offered a balanced trade-off between false positives and false negatives but had 6.0% less accuracy compared to the clinical-only model.

To improve feature extraction and minimize noise, Qiu et al. (43) presented PSFHSP-Net, a simplified neural network that utilizes an improved ResNet-18 with a single convolutional layer and three residual blocks. The model achieved strong performance, with an accuracy of 0.8995, F1-score of 0.9075, and processing speed of 65.7909 FPS, surpassing other models in efficiency. Despite a slight reduction in precision compared to ResNet-18, PSFHSP-Net significantly reduced model size from 42.64 MB to 1.48 MB, making it highly effective for real-time processing while accurately identifying PSFHSP.

In summary, the discussed studies used pre-trained deep learning architectures such as DenseNet, DeepLabV3, and YOLO. Moreover, they also used U-Net architecture for the cervix segmentation. The main focus of the above studies was the classification of maternal-fetal. However, there is still a gap in the accuracy and precision rate due to the following challenges: pre-trained models contain many learnable and gain much time in the training. In addition, the redundant information extraction took more time and reduced the classification accuracy. Hence, designing specific maternal fetal classification CNN architecture and implementing an optimization technique to reduce irrelevant features is essential.

## 3 Proposed methodology

The following section introduces the framework for classifying maternal and fetal characteristics using ultrasound images. Figure 1 showcases the framework of the proposed framework, which involves training two datasets, the fetal dataset, and FPSU23 datasets, on two models with 3 Residual blocks and 4 Residual blocks. Following this, feature extraction is performed, resulting in two feature vectors obtained from the global average pool layer. Subsequently, an improved generalized normal distribution optimization technique is employed for feature selection, and a serial probability-based approach is used to fuse the best features. As a final step, deep neural network classifiers are applied to the fused features in order to produce the final classification results.

### 3.1 Feature extraction

In this study, two datasets were used: The fetal dataset and FPSU23. The details of both datasets are given below.

#### 3.1.1 FPSU 23 dataset

**Figure 2:**
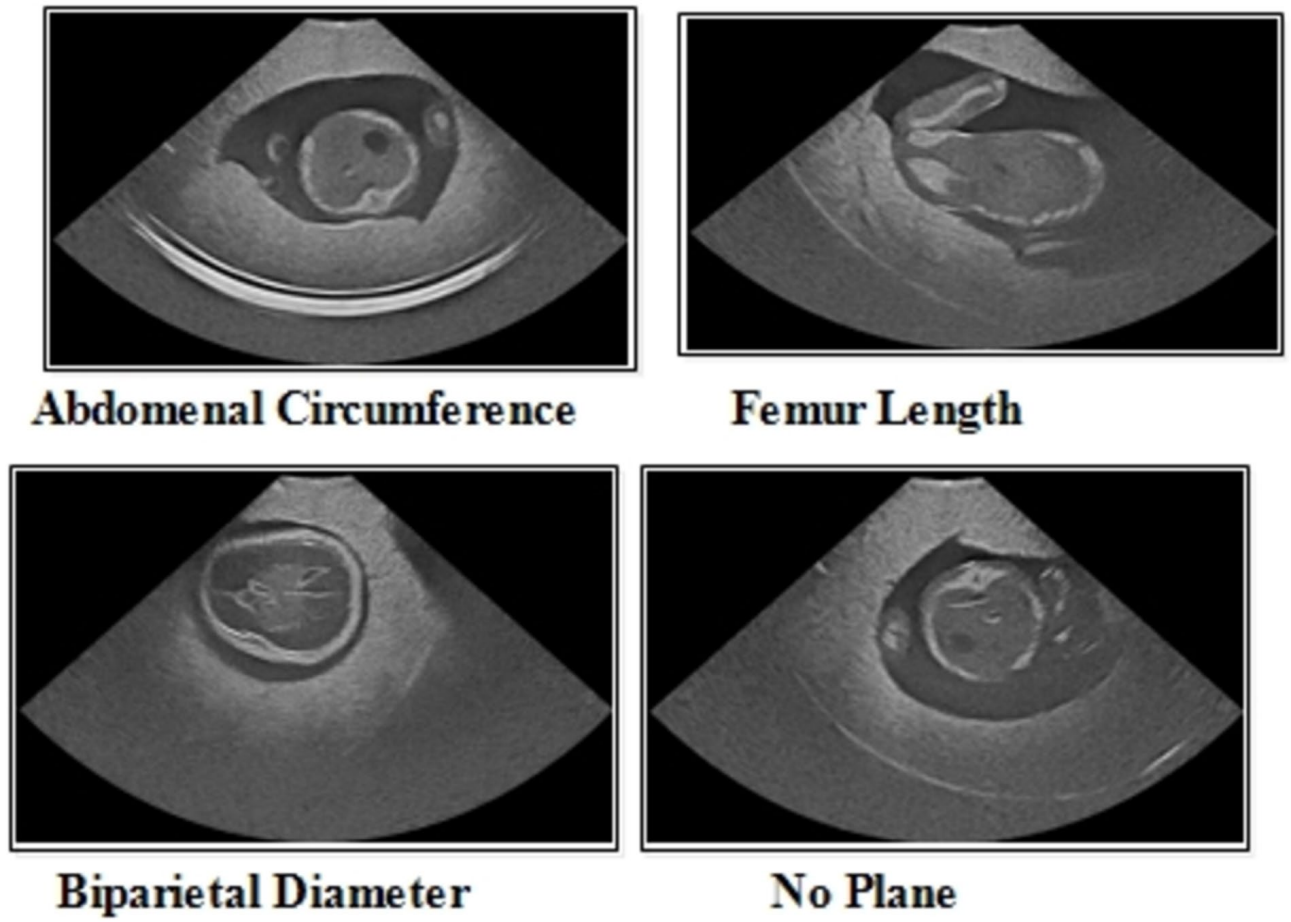
A few sample images of the FPSU23 dataset for classification purposes.

The FPSU23 was developed in 2023 and consists of four classes (18). The four classes are Abdominal Circumference (AC), Biparietal Diameter (BPD), Femur Length (FL), and No Plane. As described in Table 1, the number of images differs throughout classes, as showed in Figure 2.

**Table 1:** A summary of the FPSU23 dataset.

FPSU 23 dataset		
Class	#Images	Training/Testing
Abdominal circumference	1386	693/693
Biparietal diameter	1280	640/640
Femur length (FL)	1281	641/640
No plane	1318	659/659

*Abdominal circumference (AC):* The abdominal circumference measures the widest part of the abdomen and is used in healthcare for various purposes, including assessing adult abdominal fat and monitoring fetal growth during pregnancy.

*Biparietal diameter (BPD):* Prenatal ultrasounds utilize the biparietal diameter (BPD) measurement to assess fetal head size and development, involving the distance between major parietal bones on the fetal skull. This measurement aids in gestational age assessment during the early to mid-second trimester.

*Femur length (FL):* FL measurement in prenatal ultrasounds tracks fetal thigh bone length, assisting in gestational age estimation and growth monitoring, focusing on the second and third trimesters for gestational age determination.

#### 3.1.2 Fetal dataset

**Figure 3:**
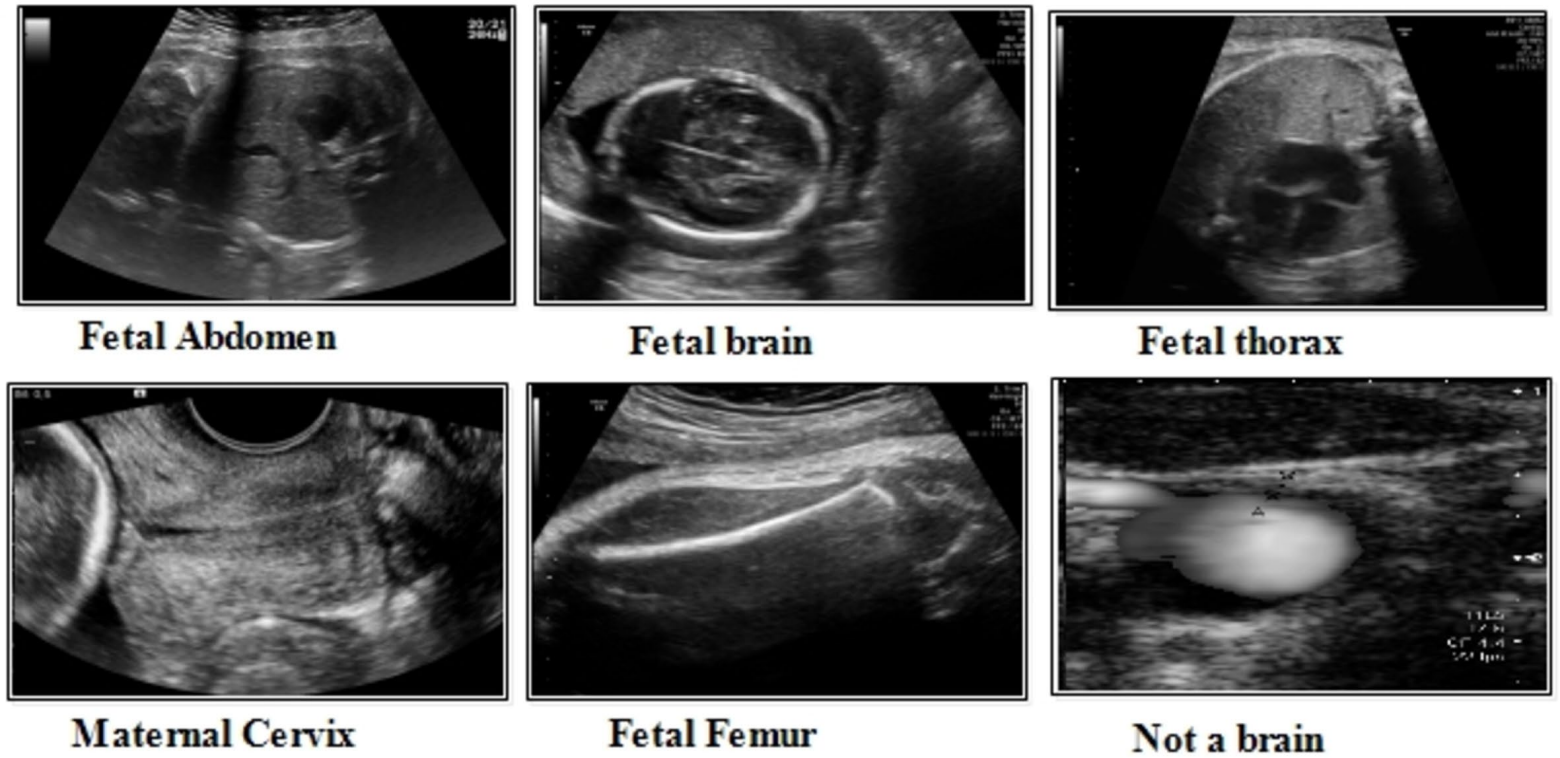
A few sample images of the fetal dataset.

This dataset includes six classes: fetal abdomen (FA), fetal brain (FB), fetal femur (FF), fetal thorax (FT), maternal cervix (MC), and not a brain (NB) (44). The fetal abdomen consists of 1422 images; Fetal Thorax contains 1718 images; fetal class includes 3092 images; maternal cervix consists of 1626 images; and the fetal femur contains 2080. As described in Table 2, the number of images differs throughout classes, as showed in Figure 3.

**Table 2:** A summary of the fetal dataset.

Fetal dataset		
Class	#Images	Training/Testing
Fetal abdomen	1422	711/711
Fetal thorax	1718	858/857
Fetal brain	3092	1546/1546
Not a brain	4213	21066/21065
Maternal cervix	1626	813/813
Fetal femur	2080	1040/1040

*Fatel brain:* As the fetal brain develops from the neural tube during pregnancy, it continues to develop into early childhood, affecting cognitive and motor development. Ultrasound and MRI are crucial tools for detecting fetal abnormalities during pregnancy.

*Fatel thorax:* An unborn infant’s fetal thorax contains respiratory and circulatory tissues from neck to abdomen. Monitoring this region can identify potential respiratory or circulatory issues and abnormalities.

*Maternal cervix:* During pregnancy and childbirth, the maternal cervix extends into the vaginal canal and plays a crucial role. For predicting premature labor, monitoring its length and condition is critical to assessing the risk of premature birth and tracking cervical changes.

### 3.2 Proposed three-residual blocks CNN

Residual blocks are a fundamental architectural component in deep neural networks, particularly in the context of convolutional neural networks (CNNs). They were introduced as part of the “ResNet” (Residual Network) architecture by Jian et al. (45). In deep networks, residual blocks address the problem of vanishing gradients, which can hinder training and limit their ability to learn complex features. In a residual block, the input to a layer is passed through that layer and directly to subsequent layers. This introduces a shortcut connection that bypasses one or more layers, allowing the network to retain and propagate gradient information more effectively during training. The motivation for choosing residual blocks because it facilitate the learning of deeper architectures by enabling the network to preserve important feature information through skip connections, ensuring better gradient flow. In the specific domain of fetal image classification, deeper networks with residual connections allow the model to capture more complex patterns without increasing the computational cost, making them highly relevant for achieving accuracy while maintaining efficiency.

The structure of a residual block typically involves three main components: the input path (the original input to the block), the residual path (which processes the input through a series of layers), and the skip or shortcut connection (which directly adds the input to the output of the residual path). The result is that a residual block aims to learn the residual (the difference between the input and the output), making it easier for the network to fine-tune the learned features. Maintaining a stride of 1 across all layers is vital in residual block to enable parallel fusion. This ensures compatibility between the sequence of various layers before addition and the layers within the block, requiring the size of the last layer in the set and the last block layer to match.

**Figure 4:**
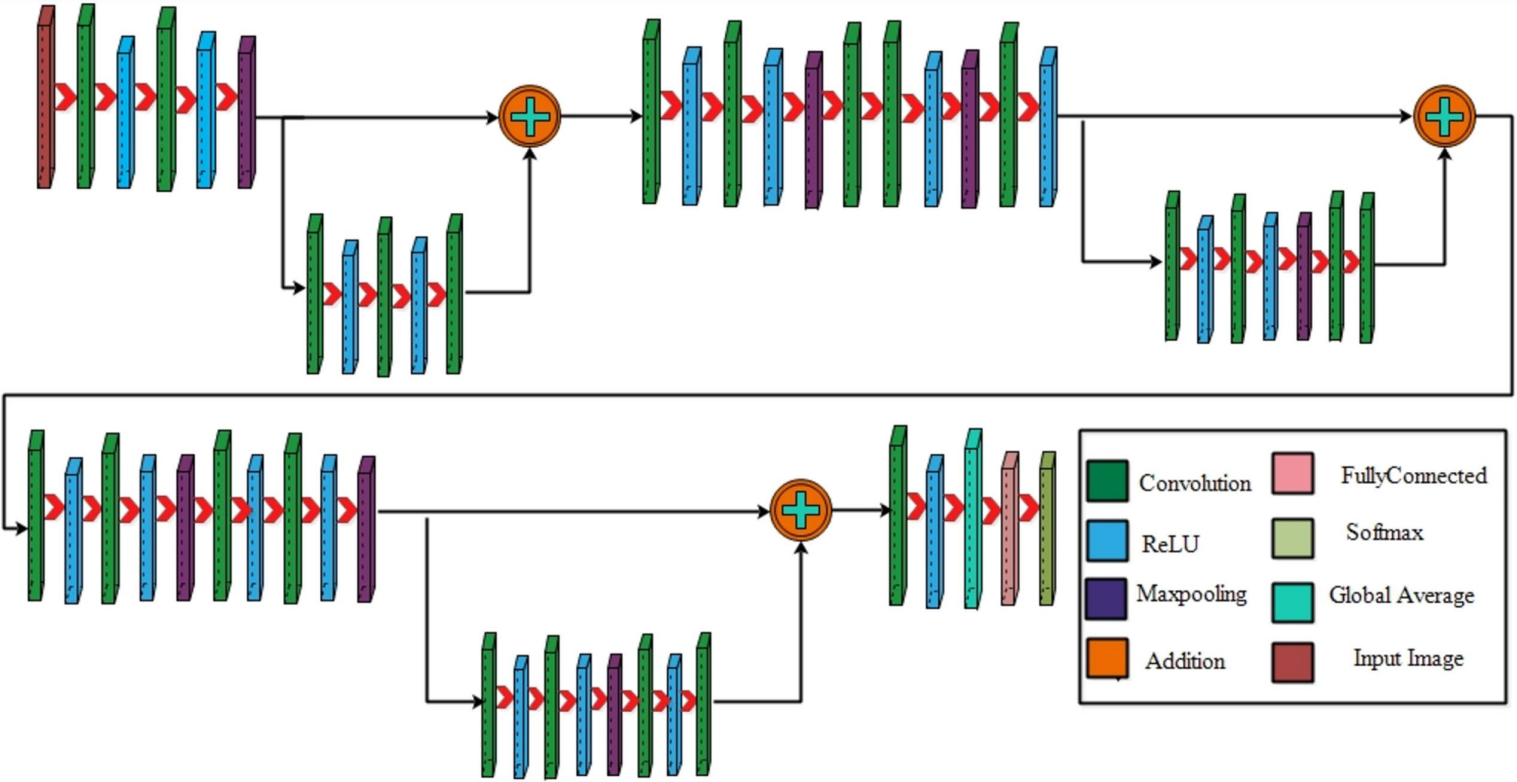
A visual architecture of 3-residual blocks CNN.

**Figure 5:**
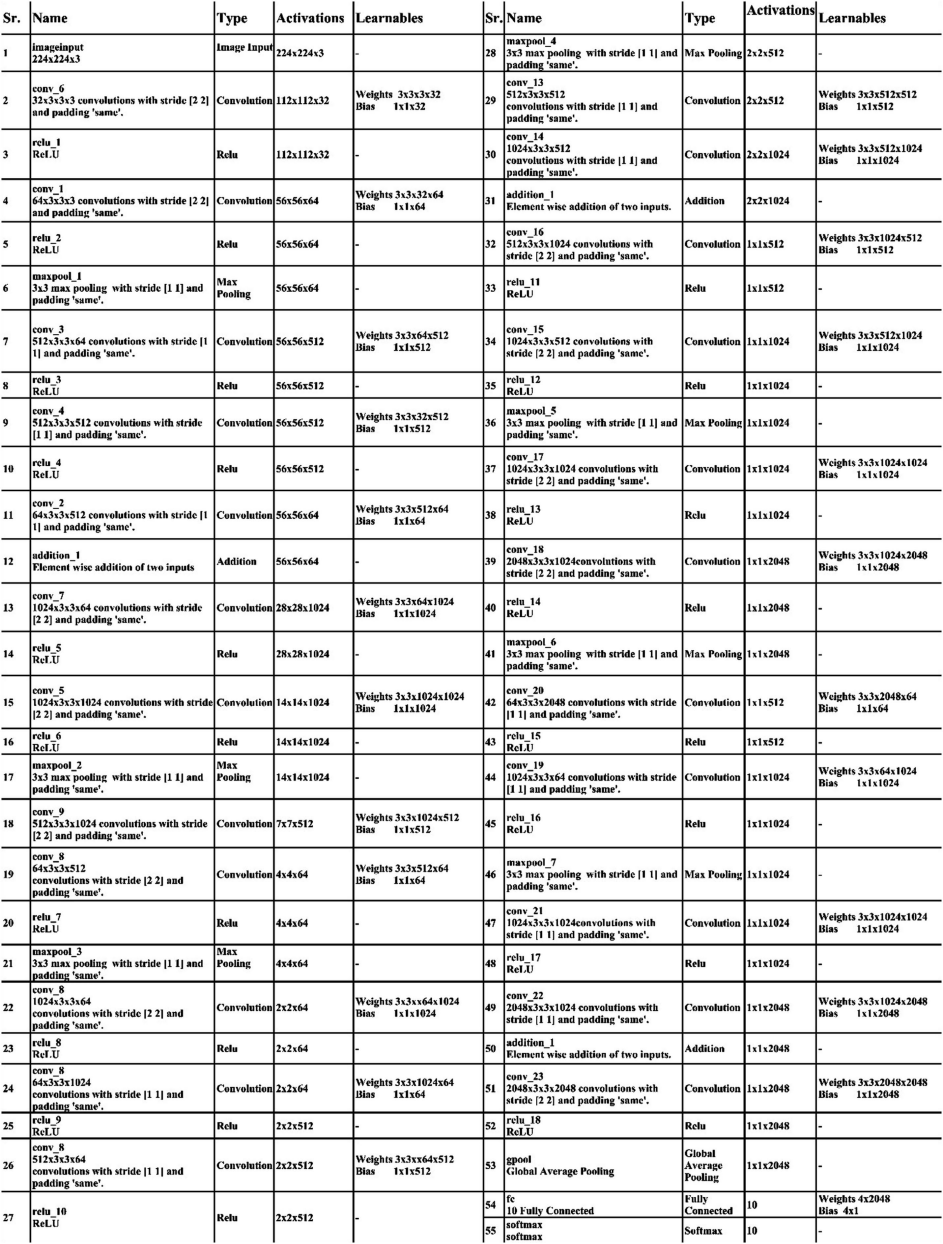
Detailed layered architecture of 3-residual blocks CNN.

**Figure 6:**
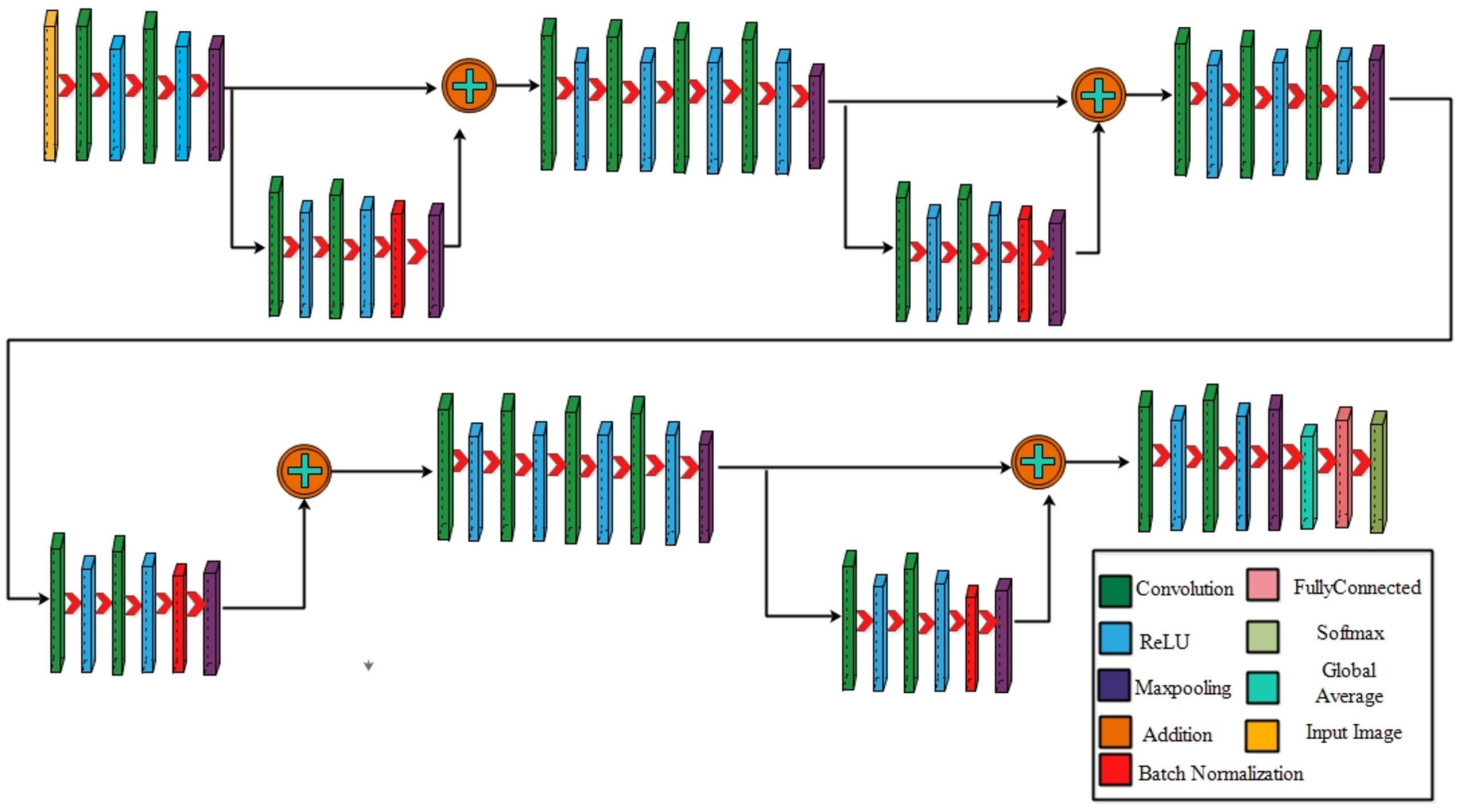
A visual architecture of proposed 4-residual blocks CNN.

**Figure 7:**
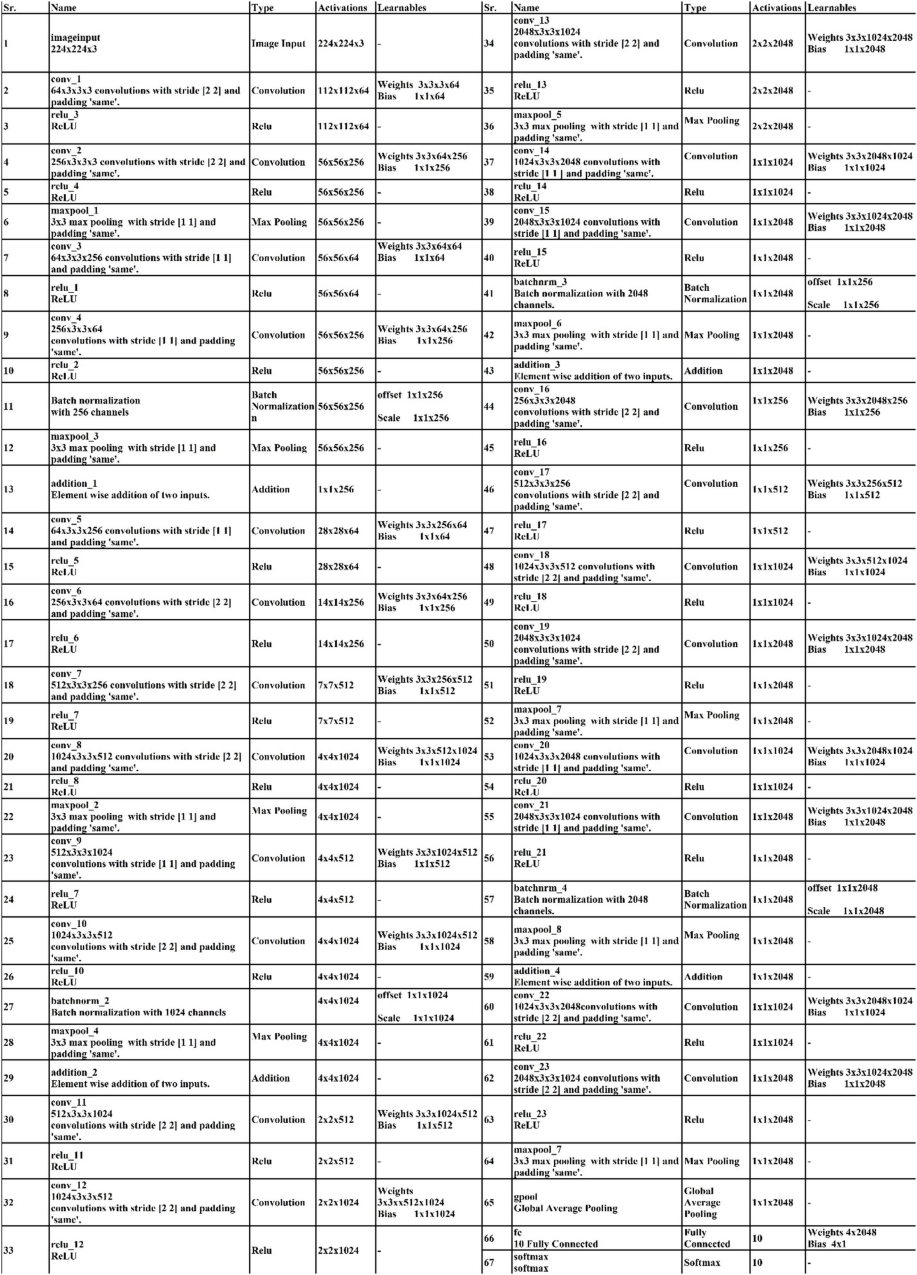
Layered architecture of the CNN with 4- residual blocks.

Initiating with an input layer sized at 224 × 224 × 2 and a depth of 3. The network begins with a convolutional layer configured with the following parameters: a depth of 32, a kernel size of 3 × 3, and a stride of 2. These values correspond to the number of filters (depth), the size of the receptive field (kernel), and the step size (stride) used to move the filter across the input. A relu activation layer is applied following each convolutional layer to introduce nonlinearity and boosting the network’s ability to recognize intricate patterns and preventing the loss of learning strength by nullifying negative values. The next step in the process is to add another convolutional layer with a depth of 64, 3 × 3 filter and a stride of 2. To reduce the spatial dimensions while preserving essential features, a Relu activation layer and a max pooling layer having 3 × 3 with stride 1 follow. Then, the first residual block is appended, consisting of five layers. The initial two convolutional layers have 512 depth, 3 × 3 filter and stride 1. A pair of relu activation layers are positioned after each convolutional layer. Concluding this block, an extra convolutional layer with a depth of 64, 3 × 3 filters, and a stride of 1 is integrated. An addition layer is introduced to establish connections, merging data through element-wise addition, thus augmenting the network’s capacity to comprehend relationships and intricacies. After this, two additional convolutions are applied, with each convolution followed by two relu activation layer. Both layers maintain a depth of 1024 and employ a filter size of 3 × 3 with a stride of 2, as shown in Figure 4. A detailed architecture is shown in Figure 5. Both layers keep a depth of 1024 and utilize a filter size of 3 × 3 with a stride of 2. A max pooling layer with a filter size of 3 × 3 and a stride of 1 is added. Subsequently, two convolutional layers are appended, one with a depth of 512 and the other with 64. Both maintain a filter size of 3 × 3 and a stride of 2. Another max pooling layer with a filter size of 3 × 3 and a stride of 1 follows. A convolutional layer and Relu layer pair are included before the subsequent residual block is added. Then a convolutional with 1024 depth, 3 × 3 filter and stride of 2 added. The second residual block is integrated in this step, featuring seven layers. The first convolutional layer, accompanied by a Relu layer, maintains a depth of 64, a filter size of 3 × 3, and a stride of 1. A pair of convolutional and Relu layers follow, both have 512 depth, 3 × 3 filter, and a stride of 1. A max pooling layer with a filter size 3 × 3 and a stride of 1 ensues. Concluding the second residual block are two convolutional layers, one with 512 depth and the other with 1024. Both layers possess 3 × 3 filter and a stride of 1. The second addition layer integrates the residual block and the layers (Figures 6, 7).

Two convolutional layers, each with a subsequent Relu layer, are implemented next. Both layers maintain a 512 depth, stride 2 and 3×3 kernel size. Following these, two convolutional with accompanying relu layers are inserted. Both layers have a depth of 1024, 3×3 filter, and a stride of 2. A max pooling layer with 3×3 filter and a stride of 1 follows. A convolutional layer, followed by a Relu layer, is then appended with a depth of 2048 and a stride of 2. Subsequently, a max pooling layer with 3×3 filter and a stride of 1 is added. The third addition layer concludes this set. The third residual block, consisting of eight layers, is introduced. The initial convolutional layer, accompanied by a relu layer, maintains a depth of 64, 3×3 filter, and a stride of 1. Following this, convolutional and Relu layers are incorporated, each holding 1024 depth, 3×3 kernel with stride 1. A max pooling layer with a filter size of 3×3 and a stride of 1 ensues. Subsequently, another pair of convolutional and Relu layers are included. The convolutional layer holds 1024 depth, 3×3 kernel size with stride 1. Concluding the third residual block is a convolutional layer with a depth of 2048, 3 × 3 kernel, with stride 1. The final step involves the addition of a pair of convolutions, each accompanied by a following relu layer, with a depth of 2048, 3 × 3 kernel, with stride 2. Lastly, the deep neural network concludes with a global average pooling layer, a fully connected layer, and a Softmax layer. In the proposed architecture, the loss was calculated using the cross-entropy loss function. This function is extensively used in classification problems since it calculates the difference between the projected probability distribution and the true labels, effectively assessing the model’s performance. The training loss for the 3-residual block model on the FPUS23 dataset was 5.7, whereas it measured 24.8 for the fetal dataset.

#### 3.2.1 Four-residual block CNN

The proposed 4-residual blocks CNN architecture consists of 4-residual blocks, with each block comprising multiple layers, and few of them are different from 3-residual blocks CNN. The first input layer of this architecture has a size of 224 × 224 ×3 and a depth of 3. A convolution layer included has 64 depth, 3 × 3 kernel size with stride 2. After convolution layers, a relu layer is incorporated; then second convolution added with 256 depth, 3×3 filter, and a stride of 2. This is succeeded by a relu layer with max pool, utilizing 3 × 3 kernel and a stride 1.

The first residual block, which has a total of six layers, is then added after that. The initial two convolutional exhibit 64 and 256 depth, both utilizing the 3×3 filter and stride value 1. Each convolutions is followed by a strategically placed set of relu layers, which introduce non-linearity into the network. A subsequent addition is a batch normalization layer boasting 256 channels. At the end of the first residual block, the addition of a max pooling layer comes into play, featuring 3 × 3 kernel with stride 1. The first addition layer combines the residual block and set of layers.

Following the initial convolution, a relu layer was added with a 64 depth size, 3 × 3 filter, with stride 2. Next, two convolutional layers were introduced, with depths of 256 and 512, using a 3×3 filter size and maintaining a stride of 2. After each convolutions, a set of relu layers was strategically placed. Another convolutional layer, followed by a relu layer, was added with a 1024 depth, 3 × 3 filter with stride 2. Lastly, a max pool added with 3 × 3 kernel.

The second residual block, which has six layers, is added at this step. The initial two convolutional layers exhibit depths of 512 and 1024, both utilizing 3 × 3 kernel with 1. Following each convolutional layer, a set of Relu layers is thoughtfully positioned to infuse non-linearity. A subsequent addition is a batch normalization layer boasting 1024 channels. At the end of the first residual block, the addition of a max pooling layer comes into play, featuring 3 × 3 kernel and stride 1. The second addition layer combines the residual block and set of layers.

After this, the first convolutional layer with the following Relu was added with 512 depth, 3 × 3 filter, and stride size 2. After this, two convolutions exhibit depths of 1024 and 2048, utilizing the same 3 × 3 filter and maintaining a stride of 2. At the end of this set, a max pooling layer has been added.

The third residual block, which has six layers, is then added. The initial two convolutional layers exhibit depths of 1024 and 2048, utilizing the 3 × 3 kernel. Following each convolutional layer, a set of Relu layers is thoughtfully positioned to infuse non-linearity. A subsequent addition is a batch normalization layer boasting 2048 channels. At the end of the first residual block, the addition of a max pooling layer comes into play, featuring 3 × 3 kernel and a stride of 1. Now, the third addition layer combines the residual block and set of layers. The 4th residual block, which has six layers, is added at this step. The initial two convolutional layers exhibit depths of 1024 and 2048, utilizing the same 3 × 3 kernel. After convolution layers, a set of relu activation is thoughtfully positioned to infuse non-linearity. A subsequent addition is a batch normalization layer boasting 2048 channels. At the end of the first residual block, the addition of a max pooling layer comes into play, featuring 3 × 3 kernel size with 4^th^ addition layer combines the residual block and set of layers.

At the end of this model, a convolution with the following relu layer added has 1024 depth, 3×3 kernel size, and stride 2. Next again, a convolution with the following rule’s has been added with 2048 depth and has same kernel. Then a max pool layer having 3 × 3 kernel added. Then, the global average pooling layer is fully connected, and the Softmax layer has been added. The training loss for the 4-residual block model on the FPUS23 dataset was 4.9, with a corresponding value of 18.8 for the fetal dataset. The proposed 3-Residual and 4-Residual-block models differ from pre-trained architectures such as ResNet18 and ResNet50 in terms of both design and parameter efficiency. 3-Residual block model has 3.77 million parameters, significantly fewer than ResNet 18’s 11.7 million parameters. It incorporates only three residual blocks, prioritizing computational efficiency without sacrificing accuracy and 4-Residual-block model has 18.87 million parameters which is fewer than ResNet50 model 23.5 million parameters. The reduced number of parameters leads to faster training and inference times, making it a lightweight alternative to deeper models.

### 3.3 Selecting hyperparameters

In this work, we use Bayesian Optimization (BO) to select hyperparameters values employed in the proposed architecture for the training process. Bayesian optimization was selected due to its efficiency in identifying optimal parameters with fewer evaluations, compared to methods such as the grid search algorithm. BO builds a probabilistic model to explore the hyperparameters space intelligently, concentrating on promising regions and avoiding exhaustive searches. BO offers a more effective solution for deep networks, where each evaluation can be computationally intensive. By contrast, grid search and other algorithms. The 3-residual-block model has been trained with 3.77 million hyper parameters, while the 4-residual-block model has been trained with 18.87 million hyper parameters. The optimization of DL architectures comprises a black box optimization process, where the objective function is a black box function. The main aim of BO is to obtain the values of hyperparameters that help improve the accuracy of training models than inexpert researchers. The process of BO is defined under the following six steps.

- By employing the Gaussian Process, the posterior distribution is adopted to update the previous results of a black box function F . Mathematically, the Gaussian Process function is defined by Equation 1.

(1) $$F\left(u\right)\sim \mathrm{GAP}\left(\mu \left(u\right),\xi \left(u,u^{\prime }\right)\right)$$

Where $\mu \left(u\right)$ denotes the mean function $\mu \left(u\right)\in R$ and covariance function k:u×u .

Using an acquisition function, the optimal point for the function F is selected using the expected improvement as the acquisition function, which is defined by Equation 2.

(2) $$EI\left(u\right)=\max\left\{0,F_{t+1}\left(u\right)-F\left(u^{+}\right)\right\}$$

- Using EI , we trained to maximize the $EI\left(u\right)$ w.r.t. current optimal value $F\left(u^{+}\right)$ . Mathematically, it is defined by Equation 3.

(3) $$u=\mathrm{argmax}\;\left(\phi \left[EI\left(u\right)\right]\right)$$

- The objective function F is utilized to find the validation set results.
- Augmenting the best-optimized sample points to the data already selected.
- The statistical Gaussian Process model is finally updated.

**Figure 8:**
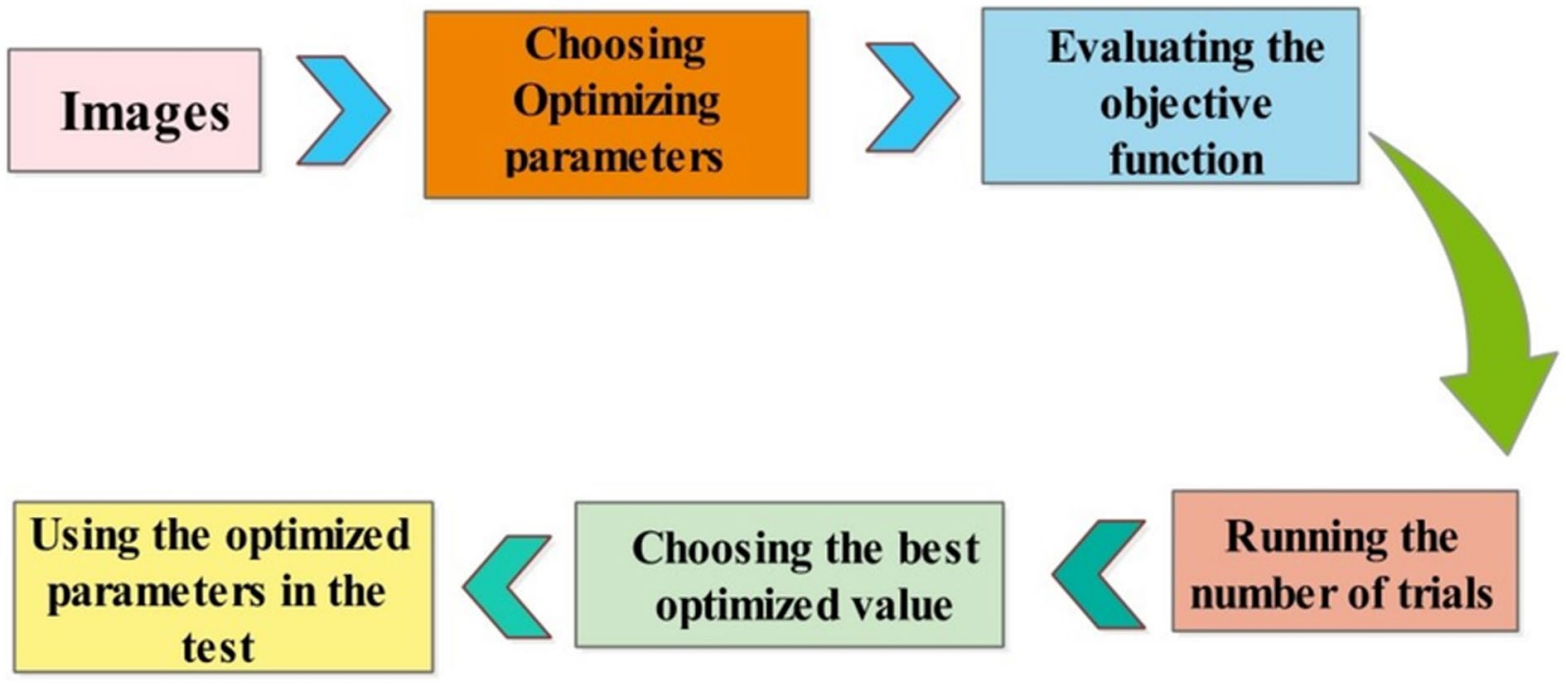
The visual process of hyperparameters selection using BO.

Iterations are repeated until a maximum number is reached. In this paper, we selected several hyperparameters after employing BO (see Table 3). In addition, the visual process of BO is shown in Figure 8.

**Table 3:** Hyperparameters selection of proposed deep models using BO.

HP Name	Range	Value
Learning rate	0.01–0.999	0.000274
Mini-batch size	16–256	128
Regularization	0.01–1	L2
Momentum	0–1	0.699
Learning algorithm	–	SGDM
L2 weight	–	1e^−6^
Activation function	–	Sigmoid

### 3.4 Training the proposed model and extracting features

On the chosen datasets, both proposed architectures have been trained using a ratio of 50 and 50 in the training process. Several hyperparameters (HPS) have been used in the training process, as mentioned in Table 3. The hyperparameters settings determine the training of the models, which is followed by the extraction of features based on the learned features. Throughout the training process, features are extracted from the global average pooling layer of the trained models. The extracted features are analyzed at this stage, and it was observed that some irrelevant information is included that should be removed for the final classification. Global average pooling layer is used for feature extraction and we obtain two feature vectors $F_{v1}$ from 3 residual block model and $F_{v2}$ from 4 residual block model of dimensions N×2048 . We proposed an improved feature selection method named the Improved Generalized Normal Distribution Optimization (IGNDO) algorithm to resolve this issue.

### 3.5 Improved feature selection algorithm and fusion

In real-world problems, population-based methods provide efficient solutions using artificial intelligence (AI) (46). Optimization techniques encompass both gradient and non-gradient approaches. In this work, we implemented an improved version of the Generalized Normal Distribution optimization called IGNDO. The improved GNDO algorithm is applied on these feature vectors separately and then obtained two best feature vectors that contain only important information (best features).

*Generalized normal distribution optimization (GNDO):* The GNDO emerges as an efficient technique without requiring fine-tuning of initial parameters. In 2020 (47), a noteworthy addition to the realm of optimization algorithms emerged as the GNDO algorithm. Various sophisticated mathematical models were used to develop this innovative algorithm, all based on the elegant concept of normal distributions. The algorithm is an example of metaheuristics, which takes inspiration from classical normal distributions. A diverse array of mechanisms is employed by the GNDO algorithm to perfectly balance the key dynamics of exploration and exploitation within its framework. This fusion of mathematical precision and creative imagination represents a significant advancement in optimization techniques. The Gaussian distribution, commonly called the normal distribution, is a fundamental tool for characterizing natural phenomena. An outstanding feature of the GNDO algorithm is its avoidance of specific controlling parameters, instead relying solely on determining essential population size and terminal condition before its execution. Furthermore, the algorithm’s simplicity shines through its uncomplicated structure, where individual positions are updated using a formulated generalized normal distribution (48).

We can define a normal distribution like this: Suppose there is a random variable y , which adheres to a probability distribution defined by the location parameter (*μ*) and the scale parameter (*δ*). The expression of its density function for probability takes the form:


(4) $$f\left(y\right)=\frac{1}{\sqrt{2\pi \delta \;}}\;\exp\left(-\frac{{\left(y-\mu \right)}^{2}}{2\delta^{2}}\right)$$

Here’s how we can describe a normal distribution: Imagine there is a random variable y , which can be denoted as a normal distribution, specifically $yN\;\left(\mu ,\delta \right)$ . As Equation 4 indicates, a normal distribution involves two key variables: the location parameter μ and the scale parameter δ. These parameters, μ and δ, represent the average value and the standard deviation of random variables, respectively.

Searching through a population-based optimization method is divided into three major stages. Initially, individuals are scattered widely. Later, they begin employing strategies that balance global and local solutions, moving toward the ideal global solution. As a result, the individuals come together to identify the best solution. This search process can be likened to the behavior of multiple normal distributions. All individuals’ positions are determined by random variables with a normal distribution. During the initial phase, there is a significant difference between the average position of individuals and the optimal solution’s location. Simultaneously, a variation in the positions of all individuals is also relatively high at this point. As the process advances into the second phase, the difference between the average and optimal solution positions systematically lessens.

**Figure 9:**
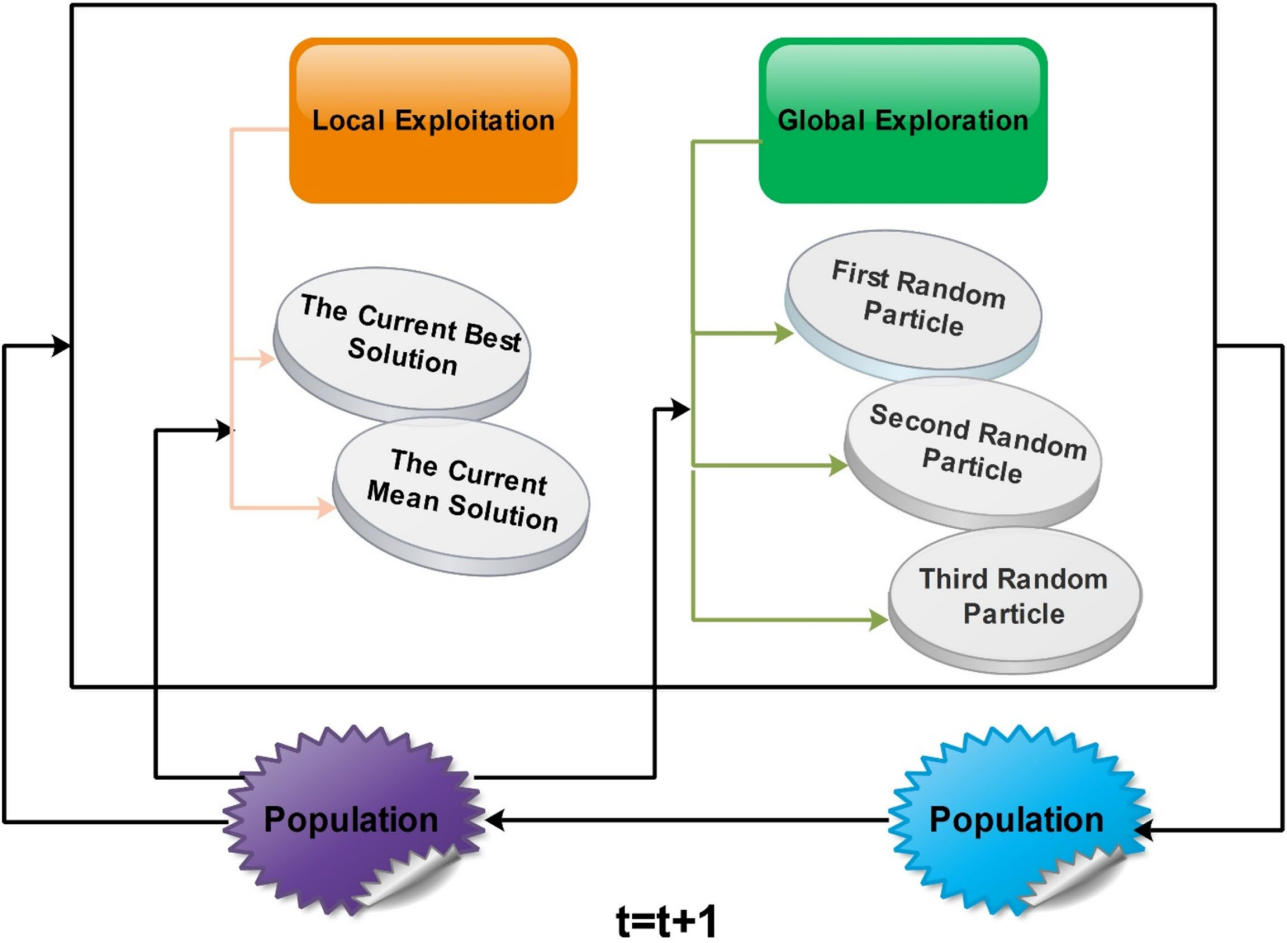
A framework of GNDO for best feature selection.

Similarly, the variability in individuals’ positions starts decreasing. In the final phase, the distance between the average position and the optimal solution’s location and the variability in individuals’ positions reach their lowest points. This signifies the population’s convergence around the most optimal solution. A visual framework of GNDO is shown in Figure 9.

GNDO features a straightforward framework in which local exploitation and global exploration represent the core information-sharing mechanisms. The execution of GNDO relies on developing local exploitation and global exploration strategies. Local exploitation revolves around a generalized normal distribution model that has been established and is directed by the current mean and optimal positions. On the other hand, global exploration involves three individuals chosen randomly. These two strategies carry equal significance in GNDO and have equal selection probabilities. GNDO stands out for its minimal requirements: specifying population size and termination conditions before starting. After evaluating individual performance using an objective function, the algorithm iterates, using random numbers to transition between exploration and exploitation until the end criterion is met. Moreover, similar to other optimization algorithms based on populations, the GNDO population is initialized using the Equation denoted as Equation 5:


(5) $$y_{i}^{t}=pj+\left(rj-pj\right)\times \lambda 5,i=1,2,3,\ldots ,N,j=1,2,3,\ldots ,D$$

Here, D represents the count of design variables, $y_{i}^{t}$ signifies the position of the ith individual at time t , the symbol pj denotes the lower limit of the jth design variable, rj signifies the upper limit of the jth design variable, and λ5 is a randomly generated number ranging between 0 and 1. The obtained outcomes are subject to analysis using a Wilcoxon signed-rank test, considering a significance level of *α* = 0.05.

#### 3.5.1 Local exploitation

Local exploitation pertains to the quest for better solutions within the immediate positions of all individuals scattered throughout the search space. To commence, the algorithm initiates by generating a random population; this is achieved by applying the subsequent Equation 6:


(6) $$w_{i}^{t}=\mu i+\delta i\times \eta \;i=1,2,3,\ldots ,N$$

The population size, denoted as N , is coupled with the trial vector $w_{i}^{t}$ for the ith individual at time t . The generalized mean position of the ith individual is represented by μi , while δi stands for the generalized standard variance. Additionally, the role of η comes into play as the penalty factor.


(7) $$\mu i=\frac{1}{3}\;\left(\;y_{i}^{t}+y_{\mathrm{Best}}^{t}+M\right)$$

(8) $$\delta i=\sqrt{\frac{1}{3}\left[\;{\left(y_{i}^{t}-\;\mu \right)}^{2}+\;{\left(y_{\mathrm{Best}}^{t}-\;\mu \right)}^{2}+\;{\left(M-\;\mu \right)}^{2}\right]}$$

(9) $$\eta =\left\{\begin{array}{c} \sqrt{-\log\left(\lambda_{1}\right)}\\ \sqrt{-\log\left(\lambda_{2}\right)} \end{array}\right.\;\begin{array}{c} \times \cos\left(2\pi \lambda_{2}\right),\mathrm{if}\;a<=b\;\\ \times \cos\;\left(2\pi \lambda_{2}+\pi \right),\mathrm{otherwise} \end{array}$$

Here, a,b,λ1,andλ2 represent random values falling within the range of 0–1 and $y_{\mathrm{Best}}$ refers to the current best position. The symbol M refers to the mean of the average position in the existing population, which can be determined by applying the following Equation 10:


(10) $$M=\frac{\sum_{i=1}^{N}y_{i}^{t}}{N}$$

It is important to acknowledge that the individual represented by ith might not always discover an improved solution through local exploitation or global exploration strategies. A screening procedure was introduced to ensure the integration of better solutions into the next generation’s population. This procedure can be formulated as follows:


(11) $$y_{i}^{t+1}=\{\begin{array}{c} v_{i,\mathrm{if}\;f\left(v_{i}^{t}\right)\;<f\left(y_{i}^{t}\;\right)}^{t}\\ y_{i}^{t},\mathrm{otherwise} \end{array}$$

#### 3.5.2 Mean position

The Generalized Mean Position (GMP) μi concept involves adjusting individuals’ positions within a population during optimization. The best individual $y_{\mathrm{Best}}$ contains valuable global solution information, and others are guided toward its direction. This can lead to premature convergence if $y_{\mathrm{Best}}$ gets trapped locally. Mean position M is introduced to mitigate this, allowing individuals to move between $y_{\mathrm{Best}}$ and M , improving solution discovery. The changing M enhances adaptability, which reduces the risk of local maxima, making it a valuable addition to local exploitation strategies.

#### 3.5.3 Standard variance

In the realm of GNDO, the infusion of the Generalized Standard Variance δi concept significantly amplifies its local search proficiency. It functions as a fluctuating sequence of randomness, directing exploration toward the Generalized Mean Position μi . The distance that exists among an individual’s location (like yt [i]) and key points like the Mean Position (M) and top performer ( $y_{\mathrm{Best}}$ ) impacts the fluctuations of this sequence. A more considerable distance leads to more substantial changes, helping struggling individuals discover better solutions. Conversely, for individuals already performing well, a gentler fluctuating sequence aids in finding even more optimal solutions nearby.

#### 3.5.4 Penalty factor

The penalty factor η contributes to the generalized standard variance’s generated randomness in the GNDO algorithm. This factor influences the resultant random sequence. Most penalty factors tend to fall between −1 and 1. It is important to emphasize that the generated generalized standard variances are consistently positive. This penalty factor’s impact expands the search directions within GNDO, thus strengthening the algorithm’s overall search capabilities.

#### 3.5.5 Global exploration

Global exploration involves searching the solution space on a broad scale to identify promising areas. In GNDO, this exploration is carried out by selecting three individuals at random, as illustrated in the following Equation:


(12) $$v_{i}^{t}=\underset{\mathrm{Local}\;\mathrm{information}\;\mathrm{sharing}}{\underset{︸}{y_{i}^{t}+ß\times \left(||ϒ3||\times v1\;\right)}}+\underset{\mathrm{Global}\;\mathrm{information}\;\mathrm{sharing}}{\underset{︸}{\left(1-ß\right)\times \left(||\;ϒ4||\times v2\;\right)}}$$

Here, ϒ3 and ϒ4 represent two random numbers following a standard normal distribution. The parameter ß denotes the adjustment parameter and takes on a random value between 0 and 1. Additionally, v1 and v2 stand for two trail vectors. Furthermore, the computation of v1 and v2 can be accomplished using the following equations:


(13) $$v_{1}=\{\;\begin{array}{c} y_{i}^{t}-y_{p1}^{t},\mathrm{if}\;f\left(y_{i}^{t}\;\right)<f\left(y_{p1}^{t}\right)\;\\ y_{p1}^{t}-y_{i}^{t},\mathrm{otherwise} \end{array}$$

(14) $$v_{2}=\{\;\begin{array}{c} y_{p2}^{t}-y_{p3}^{t},\mathrm{if}\;f\left(y_{p2}^{t}\;\right)<f\left(y_{p3}^{t}\right)\;\\ y_{p3}^{t}-y_{p2}^{t},\mathrm{otherwise} \end{array}$$

Here, p1 , p2 , and p3 represent three distinct random integers chosen from 1 to N , satisfying the condition p1≠p2≠p3≠i . By employing Equations Equation 13 and 14, the second term on the right side of Equation 12 can be termed as the “local learning term,” indicating the exchange of information between solution p1 and solution i . The third term on the right side of Equation 12 can be called “global information sharing,” signifying that individual i receives information from individuals p2 and p3 . The adjusted parameter ß is utilized to balance these two information-sharing strategies. Furthermore, ϒ3 and ϒ4 denote random numbers following a standard normal distribution, broadening GNDO’s search scope during global search operations. The absolute symbol in Equation 12 maintains consistency with the screening mechanism defined in Equations 13 and 14.

#### 3.5.6 Global search with binary cross entropy

The global search matrix is computed and later utilized to handle the uncertainty using the binary cross-entropy function. The binary cross-entropy function is defined as follows:


(15) $$CE\left(\mathrm{Gy}\right)=-\frac{1}{N}\overset{N}{\underset{i=1}{\sum }}Gy_{i}\log\left(p\left(Gy_{i}\right)\right)+\left(1-Gy_{i}\right).\log\left(1-p\left(Gy_{i}\right)\right)$$

Where Gy denotes the global search matrix obtained using Equations 12–14, the global search matrix is employed for the entropy value calculation using Equation 15. The entropy value is computed in 1, 0 form that shows the either global feature is selected (1) or not (0). The threshold function is defined as follows to get the final feature vector.


(16) $$Tr=\{\begin{array}{c} \tilde{\mathrm{Gy}}\;for\;Gy_{i}\geq CE\;\\ \tilde{\mathrm{Gy}\;}\;for\;Gy_{i}<CE \end{array}$$

Where $\tilde{\mathrm{Gy}}$ is a selected feature vector and $\tilde{\mathrm{Gy}\;}$ is a feature vector containing features that do not meet the selection criteria. A pseudo-code for the IGNDO algorithm is given in Algorithm 1. As an input, population size is defined as N , variable upper limit u and lower limit of variables l; the starting iteration count T = 0, and the maximum iteration limit Tmax all be considered. The stopping criteria of this algorithm are defined based on the number of iterations. In our case, we performed 200 iterations.

##### ALGORITHM 1 Best features selection using IGNDO algorithm

1. Using Equation 5, initialize an individual from the population.

2. Calculate the fitness value of every individual and achieve the best solution $y_{\mathrm{Best}}$ .

3. The number of iterations t is updated to *t + 1*

\*Main Loop*\

**4. While** *T < Tmax* **do**

5.**for** *i = 1 to N*

6.Generate a random value, denoted as *α*, within the range of 0 to 1.

7.**if** α *>0.5*

\*Local exploitation strategy*\

8.Equation 10 calculates the mean position M by choosing the optimal solution ybest.

9.Equations 7–9, respectively, were used to calculate the generalized mean position, generalized standard deviation, and penalty factor.

10.Computer the local exploitation strategy.

11.**else**

\*Global exploration strategy*\

12.Execute a global exploration strategy by using *Equation 11–14*

13. Find the entropy of the global search matrix using Equation 15.

14. Entropy features are passed in a final threshold function Equation 16.

15.**end if**

16.**end for**

17. Increment the current iteration number by: *t = t + 1.*

18. **end while**

**Output:** The optimal solution y best.

The above algorithm is applied separately to both proposed models’ extracted deep features. The learning rate for Stochastic Gradient Descent with Momentum (SGDM) in this work is set to 0.000274, which is a crucial parameter controlling the step size during weight updates and influencing the convergence of the model. In the end, we obtained two feature vectors of dimension N×652 and N×742, respectively. The selected features are finally fused using a simple serial-based approach and get the accuracy. The resultant serially fused vector has a dimension of N×1394 . In the final step, the fused vector is passed to the neural network classifiers to compute the final accuracy. Five neural network classifiers have been selected for the classification results as narrow-neural network (N^3^) (49), medium-neural network (MN^2^) (50), wide-neural Network (WN^2^) (51), bilayered-neural network (BN^2^) (52), and trilayered -neural network (TN^2^) (53).

## 4 Results and discussion

The results of the proposed method are discussed here with a detailed numerical analysis and confusion matrix. The results are computed on selected datasets using 10-fold cross-validation. There were two equal parts of the dataset, with 50% allocated for training and rest 50% allocated for testing. The proposed fusion architecture is trained on the fetal dataset and FPSU23 dataset; however, some hyperparameters are needed to train deep architecture and optimization algorithms. All training hyperparameters have been discussed in Table 3. The optimization algorithm selects 20 populations and 200 iterations for the best feature selection. The validation determines the best model based on performance metrics, including precision, accuracy, time, recall, FNR, MCC, F1-sc and kappa. Multiple classifiers are employed for validation, focusing on achieving the highest accuracy and minimum processing time. An MATLAB 2023b workstation equipped with 128GB RAM, a 512GB SSD, and a 12GB NVIDIA RTX 3060 graphics card was used to simulate the proposed architecture.

### 4.1 Experiments of the proposed architecture

To validate the proposed structure, a number of experiments were conducted:

Deep features extraction (54) from GAP (global average) layer of proposed 3-Residual block CNN.

- Deep features extraction from GAP (global average) layer of proposed 4-residual block CNN.
- Feature Selection (55) by employing improved GNDO for 3-Residual block deep features.
- Feature Selection by employing improved GNDO for 4-residual block deep features.
- Fusion of best-selected features using serial approach.
- Classification (56) by applying neural network classifiers.

### 4.2 FPSU23 dataset

**Figure 10:**
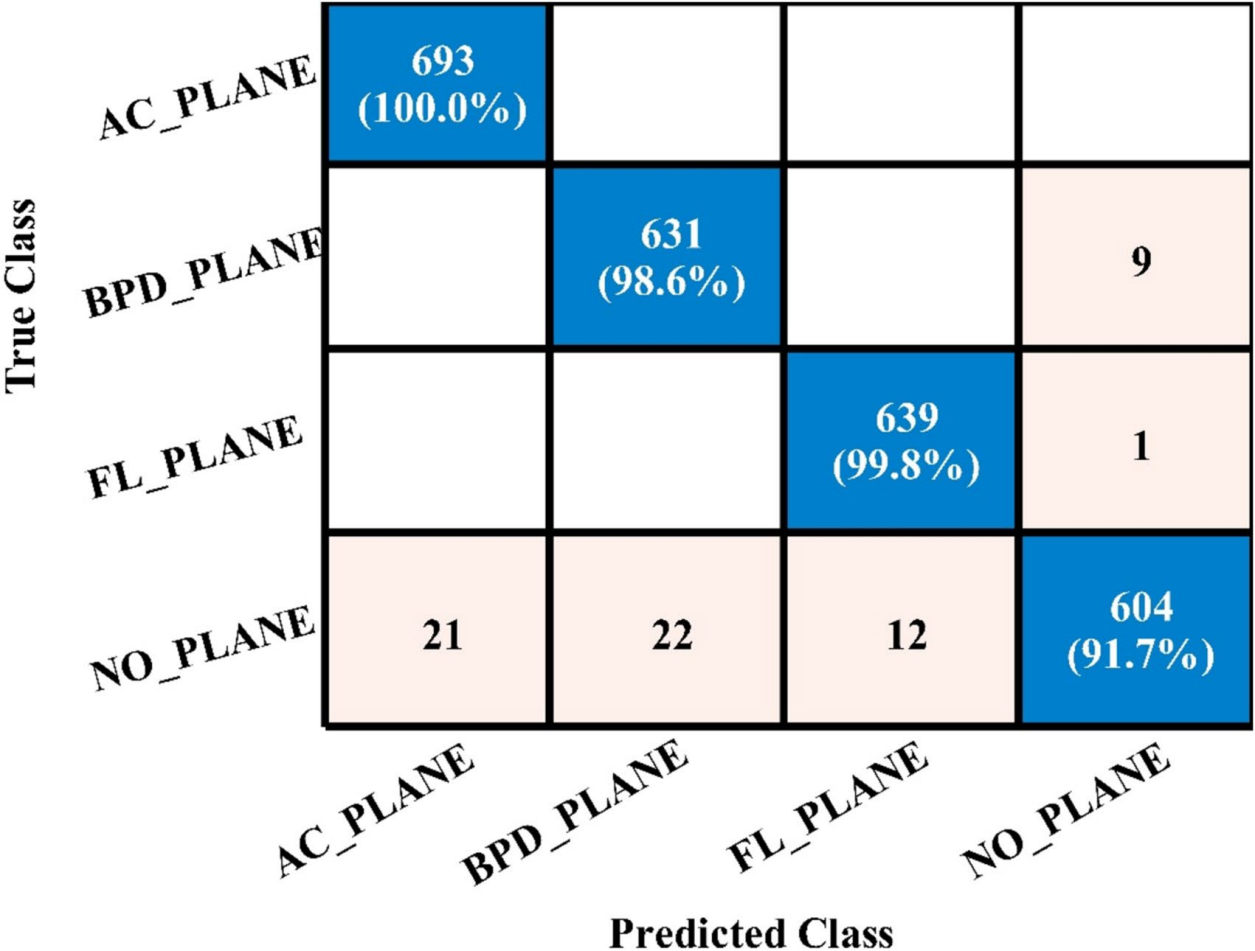
Confusion matrix of MN^2^ for 3-residual blocks CNN.

Table 4 demonstrates the classification results achieved using the 3-Residual block model on the FPSU23 dataset. The trained model’s features are extracted, and classification is performed. The MN^2^ classifier obtained the best accuracy of **97.5%**. Additional performance metrics for this classifier include 97.52 percent recall, 97.55 percent precision, 0.99% AUC and 97.53% F1-sc. The confusion matrix for MN^2^ is shown in Figure 10 that can be utilized to verify the correct prediction rate of each class. Furthermore, the confusion matrix can be used to check the computed performance metrics. During testing, 44.98 s was the lowest measured time for the MN^2^ classifier, while the TN^2^ highest recorded time is **134.97** (sec). The rest of the classifiers also obtained the accuracy values of 97.3, 97.5, 97.1, and 96.9, respectively.

**Table 4:** The 3-residual blocks CNN classification results deep features for FPSU23 dataset.

Classifiers	Accuracy %	Precision %	Recall %	F1-sc%	AUC	FNR	Time
N^3^	97.30	97.275	97.25	97.26	0.99	2.7	50.45 s
MN^2^	**97.5**	**97.55**	**97.525**	**97.53**	**0.99**	**2.4**	**44.98 s**
WN^2^	97.5	97.475	97.45	97.46	0.99	2.5	64.09 s
BN^2^	97.1	97.125	97.1	97.11	0.99	2.9	84.34 s
TN^2^	96.9	96.925	96.9	96.91	0.99	3.1	134.97 s

Bold values denotes the best results.

**Figure 11:**
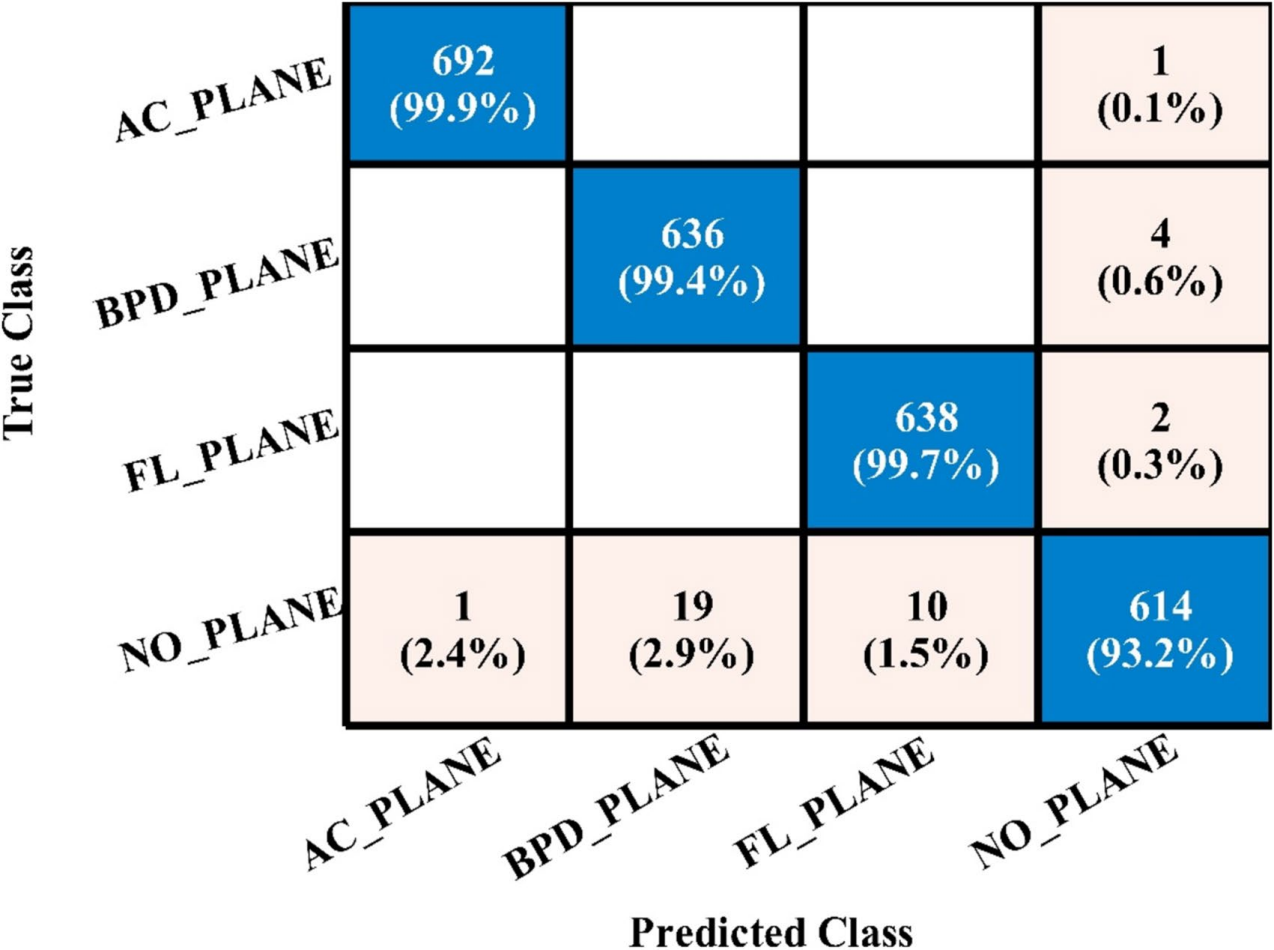
Confusion matrix of WN^2^ for 4-residual blocks CNN.

Table 5 presents the results of the proposed 4-residual block deep features for the FPSU23 dataset. The best-obtained accuracy for this model is 98.0% for the WN^2^ classifier. Also the recall is **98.05**%, precision **98.05**%, F1-sc **98.05**%, and AUC **0.99**%. Figure 11 shows the confusion matrix of this classifier that can be utilized to verify the correct prediction rate of each class. Every classifier’s computation time has also been recorded, and it is observed that the WN^2^ classifier’s least recorded time is **982.34** s, while the TN^2^ highest recorded time is **499.41** (sec). Compared to the performance of this model with 3-Residual, it is noted that the accuracy is improved, but computationally, the 3-Residual model is better. A feature selection method is implemented on both proposed models to minimize the computational time further.

**Table 5:** Classification results of 4-residual block CNN using ultrasound images.

Classifiers	Accuracy %	Precision %	Recall %	F1-sc %	AUC	FNR	Time
N^3^	97.9	97.97	99.72	98.83	0.99	0.28	406.82 s
MN^2^	97.7	97.77	97.75	97.75	0.99	2.25	477.78 s
WN^2^	**98.0**	**98.05**	**98.05**	**98.05**	**0.99**	**1.95**	**982.34 s**
BN^2^	97.9	98	97.95	97.97	0.99	2.05	485.38 s
TN^2^	97.6	97.67	97.65	97.65	0.99	2.35	499.41 s

Bold values denotes the best results.

The classification results following the implementation of an improved GNDO algorithm on 3-Residual Block CNN deep features are presented in Table 6. In this table, the best-obtained accuracy is **97.6%** for MN^2^. The values of other calculated measures include a recall rate of **97.62**%, precision rate of **97.62**%, F1 Score of **97.62**%, and AUC value of **0.99**%, respectively. Comparing the results of this experiment with Table 4, it is observed that the accuracy is improved, and time is significantly reduced. The MN^2^ classifier’s least recorded time was 20.57 s after selecting the best features and 44.98 s before the selection process. A proposed classification method result is presented in Table 7 after using the improved GNDO algorithm on 4-residual blocks CNN deep features. After employing this experiment, the best-obtained accuracy of 98.0% has been achieved for N^3^. The recall is **97.75**%, precision **98.05**%, F1-sc of **97.89**%, and AUC value of **0.99**% obtained for this classifier are better than the performance noted in Table 5. The computational time is also reduced for this experiment, and it is observed that the overall accuracy is also improved, which shows the strength of this method. The N^3^ classifier’s least recorded time is **49.08** s after selecting the best features.

**Table 6:** Feature selection by improved GNDO algorithm of 3-residual blocks model using FPSU23 dataset.

Classifiers	Accuracy %	Precision %	Recall %	F1-sc %	AUC	FNR	Time
N^3^	97.6	97.55	97.6	97.57	0.99	2.45	20.77 s
MN^2^	**97.6**	**97.62**	**97.62**	**97.62**	**0.99**	**2.38**	**20.57 s**
WN^2^	97.5	97.47	97.47	97.47	0.99	2.53	32.47 s
BN^2^	97.2	97.12	97.17	97.14	0.99	2.88	48.05 s
TN^2^	97.0	97.02	97.07	97.04	0.99	2.98	78.87 s

Bold values denotes the best results.

**Table 7:** Feature selection by improved GNDO algorithm of 4-residual blocks model using FPSU23 dataset.

Classifiers	Accuracy %	Precision %	Recall %	F1-sc	AUC	FNR	Time
N^3^	**98.0**	**97.75**	**98.05**	**97.89**	**0.99**	**2.25**	**42.21 s**
WN^2^	98.0	98.02	97.95	97.98	0.99	1.975	50.01 s
MN^2^	98.0	98.02	98.02	98.02	0.99	1.975	90.82 s
BN^2^	97.2	97.15	97.15	97.15	0.99	2.85	44.59 s
TN^2^	96.9	96.92	96.92	96.92	0.99	3.08	49.08 s

Bold values denotes the best results.

**Figure 12:**
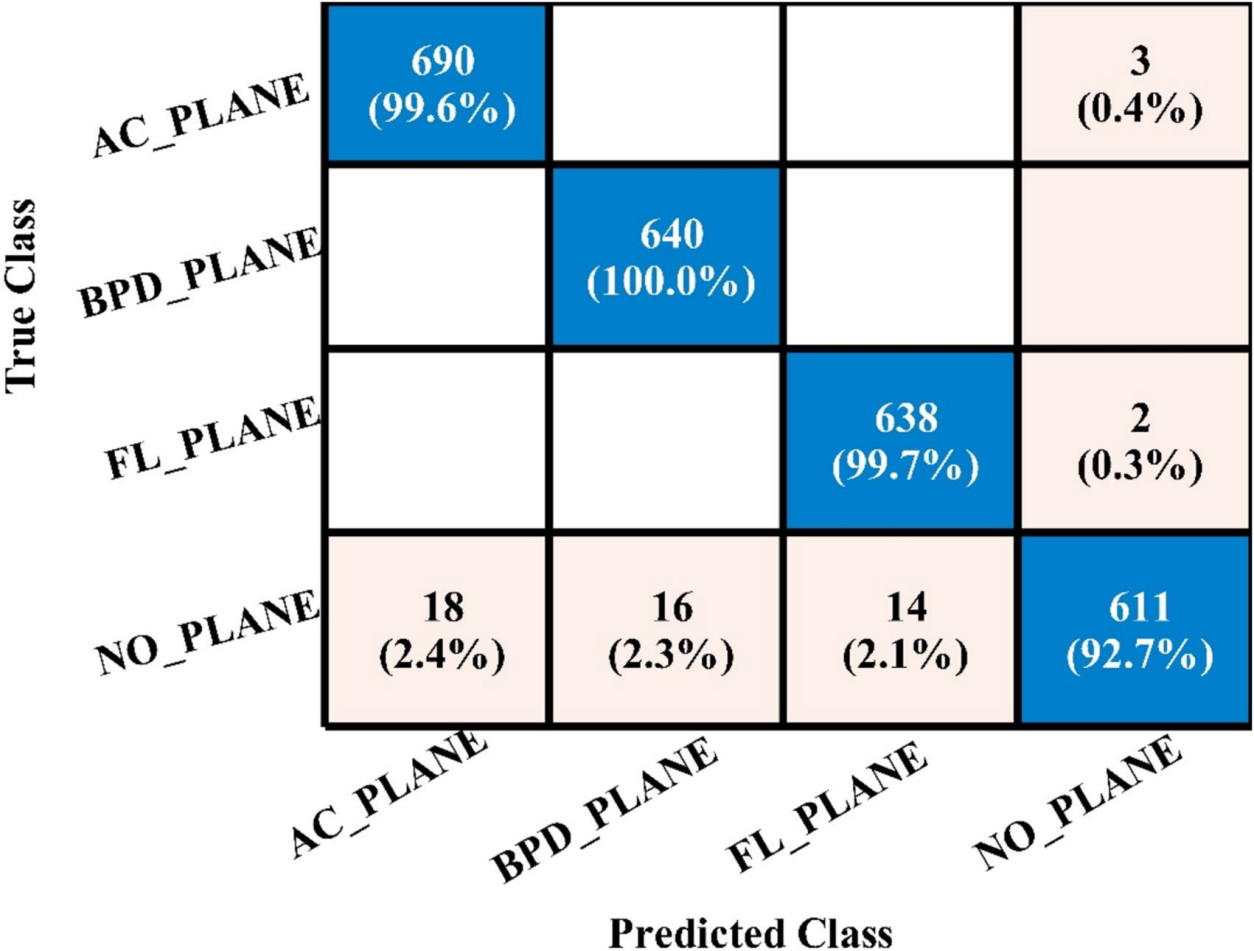
MN^2^ confusion matrix after the fusion of best features for FPSU23 dataset.

Ultimately, the optimal characteristics from both models were combined, and Table 8 displays the outcomes. With respect to recall, precision, and F1 score, MN2 yielded the best accuracy of 98.5, 98.52, 98.55, and 98.53%, respectively. Improved accuracies of 98.1, 98.3, 98.1, and 98.4% were attained by the remaining classifiers. The confusion matrix of MN2 following the fusion procedure is displayed in Figure 12, which can be used to confirm the overall performance metrics. While the first experiment’s maximum accuracy was 97.5%, the second experiment’s maximum accuracy was 98.0%, the third experiment’s maximum accuracy was 97.6%, the fourth experiment’s maximum accuracy was 98%, and the fifth experiment’s maximum accuracy was 98.5%. Hence, overall, the proposed framework and the optimization process show improvement after the fusion process.

**Table 8:** Classification results of the FPSU23 dataset after the fusion of best selected features.

Classifiers	Accuracy %	Precision %	Recall%	F1-sc%	AUC	FNR	Time
N^3^	98.1	98.15	98.175	98.16	0.99	1.85	74.036 s
MN^2^	**98.5**	**98.52**	**98.55**	**98.53**	**0.99**	**1.48**	**49.939 s**
WN^2^	98.3	98.3	98.3	98.3	0.99	1.7	88.82 s
BN^2^	98.1	98.1	98.15	98.12	0.99	1.9	83.12 s
TN^2^	98.4	98.45	98.47	98.45	0.99	1.55	131.73 s

Bold values denotes the best results.

### 4.3 Fetal dataset results

The classification results of the fetal dataset using the proposed architecture have been presented in this subsection. The proposed results are computed in several experiments, as discussed in the above section. The results of the first experiment are presented in Table 9, showing the maximum accuracy of **78.3**% for the WN^2^ classifier. Other measures are recall **77.75**%, precision 77.28%, the F1-sc 77.51%, kappa 0.1922, and MCC **0.7194**, respectively. The rest of the classifier obtained an accuracy of 76.0, 77.1, 75.9, and 75.2%, respectively. Computationally, this architecture is a little expensive, as the minimum noted time is 268.29 (sec). Table 9 (2nd quarter) illustrates the classification results for the proposed 4-residual blocks CNN with deep features. The best-obtained accuracy of this experiment is **84.1**% for the MN^2^ classifier. The kappa and MCC measures are also computed, and the obtained values are 0.4255 and 0.7891. The rest of the classifiers obtained accuracy values of 83.3, 83.9, 83.5, and 83.6%, respectively. The MN^2^ achieved a high accuracy but consumed 1230.8 (sec) for the execution.

**Table 9:** Classification results of proposed architecture employing fetal dataset.

Classifiers	Accuracy%	Recall %	Precision %	Kappa	MCC	F1-sc %	AUC	FNR	Time
Classification results of the proposed CNN utilizing deep features with three residual blocks									
N^3^	76.0	75.65	74.96	0.1360	0.7029	75.30	0.897	24.35	483.5 s
MN^2^	77.1	76.33	70.03	0.1772	0.7147	73.04	0.903	23.67	268.29 s
WN^2^	**78.3**	**77.75**	**77.28**	**0.1922**	**0.7194**	**77.51**	**0.903**	**22.25**	**511.46 s**
BN^2^	75.9	75.35	74.91	0.1329	0.7010	75.12	0.892	24.65	469.5s
TN^2^	75.2	74.48	74.05	0.1070	0.6909	74.26	0.886	25.52	449.25 s
Classification results of the proposed CNN utilizing deep features with four residual blocks									
N^2^	83.3	81.73	81.03	0.3996	0.7797	81.37	0.95	18.27	1503.1 s
MN^2^	**84.0**	**82.28**	**81.88**	**0.4225**	**0.7891**	**82.07**	**0.94**	**17.72**	**1230.8 s**
WN^2^	83.9	82.53	81.95	0.4215	0.7894	82.23	0.94	17.47	1736.8 s
BN^2^	83.5	82.55	81.33	0.4067	0.7842	81.93	0.95	17.45	1400.2 s
TN^2^	83.6	82.06	81.18	0.4087	0.7825	81.61	0.95	17.94	1325.7 s
The proposed 3-residual blocks CNN deep features’ classification results are presented after using an improved GNDO method									
N^2^	74.9	74.53	73.7	0.016	0.6896	74.11	0.895	25.47	326.65 s
MN^2^	76.2	75.7	75.35	0.1745	0.7145	75.52	0.905	24.3	206.37 s
WN^2^	**78.8**	**78.21**	**78.01**	**0.2074**	**0.7269**	**78.10**	**0.914**	**21.79**	**291.13 s**
BN^2^	75.2	74.36	73.81	0.1065	0.6893	74.08	0.889	25.64	319.77 s
TN^2^	74.8	74.15	73.31	0.0926	0.6849	73.72	0.889	25.85	333.8 s
The proposed 4-residual blocks CNN deep features’ classification results are presented after employing an improved GNDO method									
N^3^	**83.8**	**82.51**	**81.71**	**0.4179**	**0.7879**	**82.10**	**0.96**	**17.49**	**198.56 s**
WN^2^	83.5	82.03	81.33	0.4052	0.7833	81.67	0.95	17.97	240.41 s
MN^2^	83.7	82.51	81.65	0.4138	0.7872	82.07	0.94	17.49	480.48 s
BN^2^	83.3	81.61	80.88	0.3970	0.7781	81.24	0.95	18.39	200.17s
TN^2^	83.4	81.73	81.03	0.4042	0.7798	81.37	0.95	18.27	206.69 s
Classification outcomes for the proposed architecture incorporating feature fusion									
N^2^	87.6	86.55	86.33	0.5543	0.8383	86.43	0.94	13.45	139.98 s
MN^2^	87.9	86.71	86.7	0.5649	0.8422	86.70	0.966	13.29	77.109 s
WN^2^	**88.6**	**87.28**	**87.41**	**0.5849**	**0.8499**	**87.34**	**0.969**	**12.72**	**130.62 s**
BN^2^	87.1	85.68	85.75	0.0.5370	0.8306	85.71	0.93	14.32	262.03 s
TN^2^	87.1	85.65	85.66	0.5349	0.8305	85.65	0.93	14.35	314.5 s

Bold values denotes the best results.

**Figure 13:**
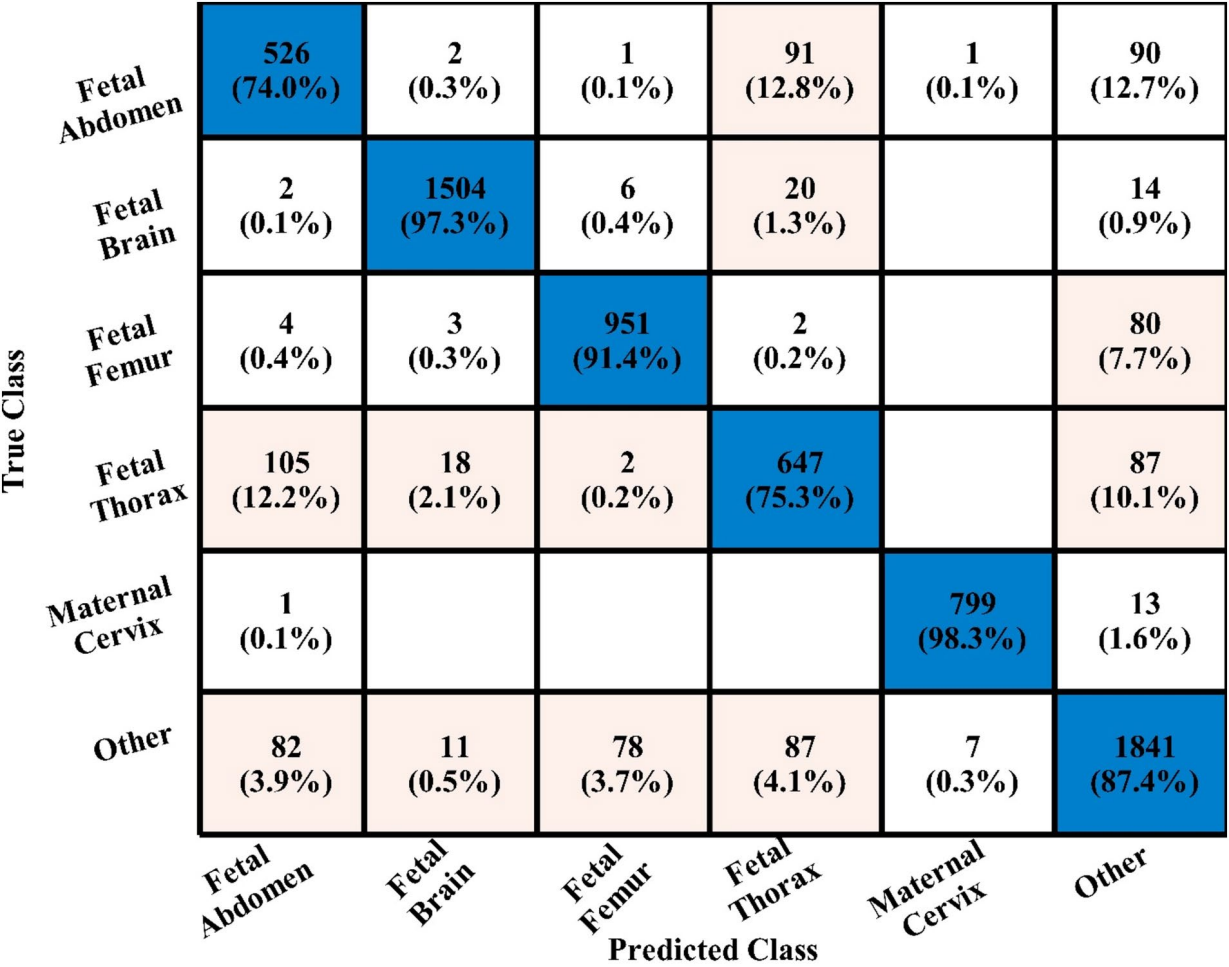
WN^2^ classifier confusion matrix after the fusion process using fetal dataset.

In the third experiment, the feature selection algorithm is applied using three residual blocks CNN deep features, the maximum accuracy was obtained of 78.8% for WN^2^ classifier. The other computed measures of WN^2^ classifier, such as recall 78.21%, precision 78.01%, kappa value of 0.2074, MCC value of 0.7269, F1-sc of 78.10%, and AUC value of 0.914%, respectively. The computation time for each classifier is also determined, and MN^2^ is executed in the maximum processing time of 291.13 s, whereas the minimum noted time is 206.37 s for WN^2^. Similarly, the feature selection algorithm is applied on 4-residual blocks deep features and obtained an accuracy of 83.8% for N^3^. This classifier also has other parameters that have been computed, such as a recall rate of 82.51%, precision rate of 81.71%, kappa measure of 0.4179, MCC measure of 0.7879, F1 Score of 82.10%, and AUC 0.96%, respectively. The Narrow Neural Network classifier’s least recorded time is 198.56 s, while the TN^2^ greatest recorded time is 480.48 (sec). The classification results of the proposed 3- and 4-residual block CNN deep features are shown at the end of Table 9. This table presented the best-obtained accuracy of 88.6% for WN^2^ classifier. The other calculated measures were recall rate of 87.28%, precision rate of 87.41%, kappa value of 0.5849, MCC value of 0.8499, F1-sc 87.34%, and AUC values of 0.96, respectively. Furthermore, the WN^2^ confusion matrix is shown in Figure 13 that shows the correct prediction rate of each class (Tables 10–13).

**Table 10:** Classifiers of FPSU23 dataset with their accuracies.

Classifiers	3-residual block	4-residual block	GNDO 3-residual block	GNDO 4-residual block	Fusion
Medium NN	97.5	97.7	97.6	98.0	98.5
Tri-layered NN	96.9	97.6	97.0	96.9	98.4
**Diff**	**0.6**	**0.1**	**0.6**	**1.1**	**0.1**

Bold values denotes the best results.

**Table 11:** Classifiers of fetal dataset with their accuracies.

Classifiers	3-residual block	4-residual block	GNDO 3-residual block	GNDO 4-residual block	Fusion
Medium NN	78.3	83.9	78.8	83.5	88.6
Tri-layered NN	75.2	83.6	74.8	83.4	87.1
**Diff**	**3.1**	**0.3**	**4**	**0.1**	**1.5**

Bold values denotes the best results.

**Table 12:** Accuracy comparison between the proposed method and existing methods.

Authors/Reference	Dataset		Accuracy (%)	Time (sec)
	Fatel plans	FPSU23		
Prabakaran et al. (18)		✓	88.0	–
Our proposed 3- residual blocks CNN.	✓		**88.6**	130.62 s
Our proposed 4-residual blocks CNN.		✓	**98.5**	c

Bold values denotes the best results.

**Table 13:** Comparison of the two proposed architectures utilizing the fetal plans dataset with a number of other cutting-edge optimization algorithms.

Methods	Common maternal fetal planes	Time (sec)
**Proposed 3-RB Model + IGNDO**	**78.8**	**291.13 s**
**Proposed 4- RB Model + IGNDO**	**83.8**	**198.56 s**
Proposed + GA	74.6	92.990
Proposed + PSO	75.1	91.643
Proposed + WOA	75.6	103.563
Proposed + BCO	76.3	111.336
Proposed + FA	77.8	95.245
Proposed + ACO	76.5	110.511
Proposed+ lion optimization	79.6	121.6779

Bold values denotes the best results.

### 4.4 Discussion

A detail discussion of the proposed method results have been presented in this section. Initially, the t-test has been performed to validate the performance of the selected classifiers. The t-test, a statistical analysis method, is utilized to assess whether there is a significant difference between the means of two groups. When the experiments were finished, the Student’s t-test was used to examine the findings. Two classifiers were first chosen as they both showed constant accuracies during every experiment. This selection made it possible for us to do the experiment using both a low- and high-accuracy classifier, allowing for a comparative comparison. Our initial hypothesis $h_{o}$ = the accuracy of the chosen classifiers does not vary significantly in a meaningful way. A *t*-test for the FPSU23 Dataset compared two classifiers: Medium NN and tri-layered NN. The corresponding accuracy of these classifiers is presented in Table 14. For fetal dataset these two classifiers compared: wide NN and tri-layered N^2^. The corresponding accuracy of these classifiers is presented in Table 14.

**Table 14:** Comparison of both proposed architectures with several other state-of-the-art optimization algorithms using FPSU23 dataset.

Methods	Common maternal fetal planes	Time (sec)
**Proposed 3-RB Model + IGNDO**	**97.6**	**20.7 s**
**Proposed 4- RB Model + IGNDO**	**98.0**	**42.21 s**
Proposed + GA (30)	93.4	76.2234
Proposed + PSO (31)	94.1	54.0953
Proposed + WOA	94.9	67.1674
Proposed + BCO	94.5	81.5395
Proposed + FA	96.3	71.5572
Proposed + ACO	95.2	61.4463
Proposed+ lion optimization	96.8	58.4460

Bold values denotes the best results.

The difference in their accuracies is calculated using the following formula, where in Equation 17, the term $Acc\left(C1\right)$ stands for the uppermost accuracy, and $Acc\left(C2\right)$ stands for the lowermost accuracy. The Mean μ is evaluated using Equation 18, which is 0.5 for the FPSU23 Dataset and 1.8 for the Fetal Dataset. In this Equation, the N denotes a total number of methods.


(17) $$\mathrm{Diff}=|Acc\left(C1\right)-Acc\left(C2\right)|$$

(18) $$\mathrm{Mean}=\mu =\frac{1}{N}\overset{N}{\underset{i=1}{\sum }}\left(Diff_{i}\right)$$

(19) $$\mathrm{Standard}\;\mathrm{Deviation}=\sigma =\sqrt{\frac{\sum_{i=1}^{N}{\left(Diff_{i}-\mu \right)}^{2}}{N-1}}$$

After that, the standard deviation is computed using the formula shown in Equation 19, and the obtained value is 0.4183 for the FPSU23 Dataset and 1.865 for the Fetal Dataset. After calculating the t-selection value using Equation 20, we found it to be 2.6728 for FPSU23 and 2.15806 for Fetal dataset. These values are used as reference points for conducting the Student’s *t*-test.


(20) $$t-\mathrm{selection}=t=\frac{\sqrt{N}\times \mu }{\sigma }$$

The degrees of freedom are computed as degreeoffreedom=df=N−1 . Assuming the null hypothesis is true at a significance level of 0.05, there is a 5% probability of achieving the observed results, corresponding to a *p*-value of 0.05. We calculated the confidence interval using the t-test table, considering the p-value and degrees of freedom, resulting in the interval (−2.776, +2.776). The computation of this confidence interval follows the Equation (21).


(21) $$\mathrm{Confidence}\;\mathrm{Interval}=I=\left[\frac{-t_{p}}{2},\;N-1,\frac{t_{p}}{2},\;N-1\right]$$

There is no discernible difference between the two chosen classifiers’ accuracies, as the t-selection value falls within the range based on this confidence interval. Therefore, our hypothesis is accepted.

#### 4.4.1 Comparison with neural nets and SOTA

**Figure 14:**
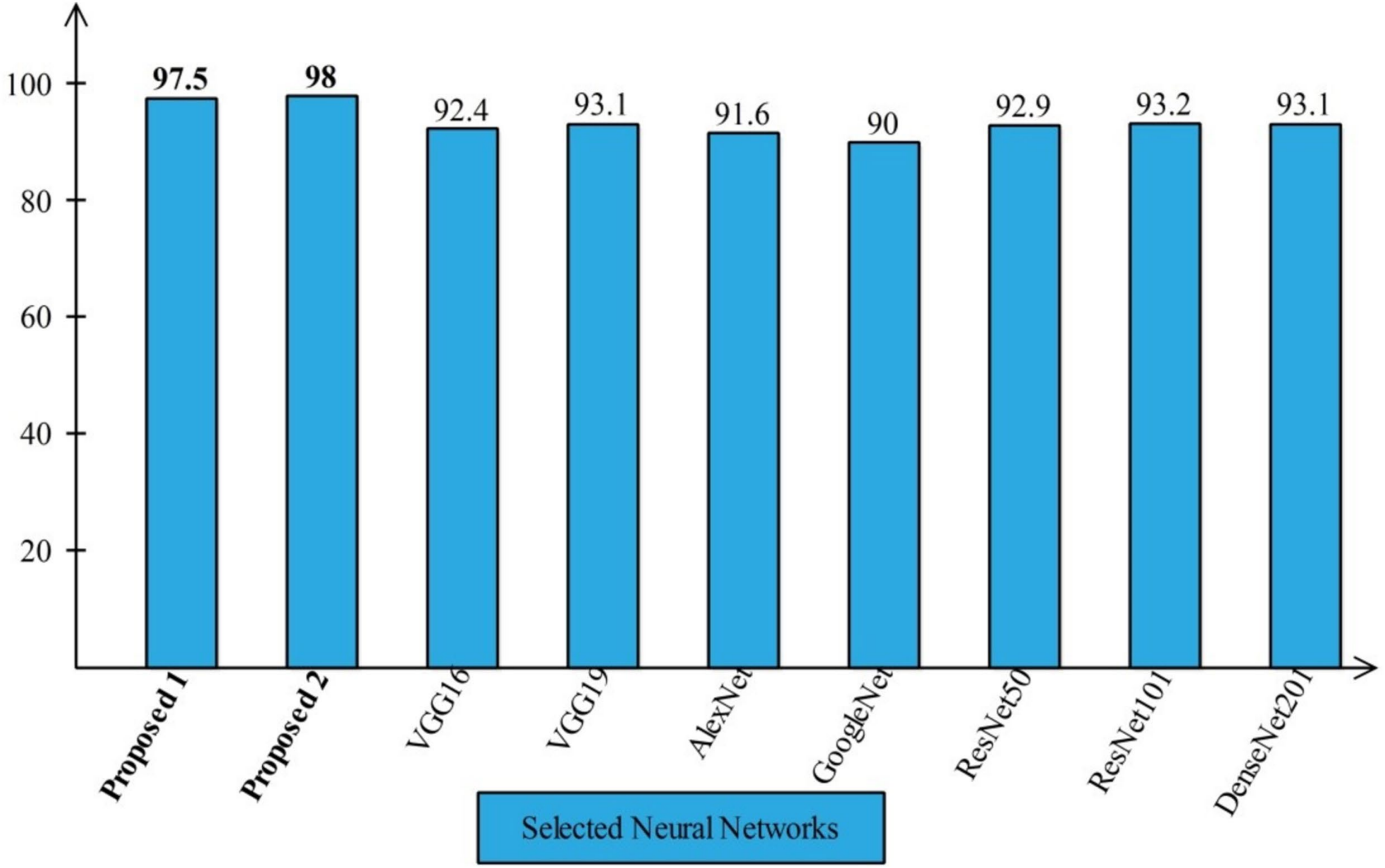
Comparison in term of accuracy for FPSU23 dataset by using several neural networks.

**Figure 15:**
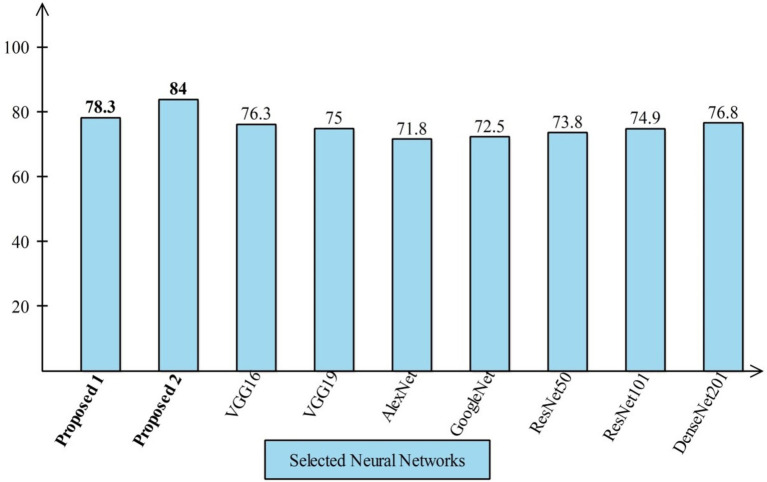
Comparison in term of accuracy for maternal fetal dataset by using several neural networks.

Several state-of-the-art (SOTA) recent deep learning architectures are compared for the two chosen datasets. The methods selected for the comparison is VGG16, VGG19, AlexNet, GoogleNet, ResNet50, ResNet101, DenseNet201, and MobileNet-V2. A comparison is plotted in Figures 14, 15. In Figure 14, the comparison is performed for the FPSU23 dataset that shows the maximum accuracy is obtained by proposed 2 (4-Residual). The maximum accuracy obtained by other pre-trained models is 93.2% of Resnet101. Figure 15 compares the maternal fetal dataset, showing that the proposed 2 (4-Residual) obtained the highest accuracy of 84%. In addition, the authors of the work (18) used the FPSU23 dataset and obtained a maximum accuracy of 88%. However, the proposed method improved overall accuracy of 88.6 and 98.5%, respectively.

#### 4.4.2 Comparison with other optimization algorithms

An analysis of the improved GNDO in contrast to a few other optimization algorithms inspired by nature. The proposed architecture 3 residual block model obtained the highest accuracy of 78.8% and 4 residual block model 83.8% using the IGNDO algorithm for common maternal fetal planes, as shown Table 13 respectively. The accuracy of the proposed architecture’s GA-based feature selection was 74.6%. A 75.1% accuracy rate was achieved for PSO-based feature selection. The accuracy of the proposed architecture when combined with the Whale optimization algorithm (WOA) is 75.6%. With additional optimization, the proposed approach yielded an accuracy of 79.6%. The third highest accuracy for this dataset is 77.8 using the paired of firefly algorithm. Hence, this table demonstrates that the proposed architectures performed effectively with IGNDO using Fetal Planes optimization algorithm. Similarly, Table 14 presents the results of FPSU23 dataset and obtained the improved performance for proposed IGNDO optimization algorithm.

## 5 Conclusion

Automatic classification of common maternal fetuses from ultrasound images has been presented in this work using information fusion of two novel deep learning architectures. Two publicly available datasets have been employed in this work to train and test the proposed framework. Two deep learning architectures have been proposed, and the hyperparameters selected using Bayesian Optimization (BO) to improve the efficiency of proposed architectures in training phase. The extracted features from GAP layers of both trained CNN architectures have been optimized utilizing an improved version of the GNDO. The optimal features are combined into a single vector using a serial-based approach, which is then fed into neural networks for the final classification. The proposed method obtained an improved accuracy of 88.6 and 98.5% on the selected datasets compared to SOTA techniques. Therefore, the proposed architecture is suitable for efficiently and effectively classifying common maternal fetuses using ultrasound images. In addition, the proposed framework is effective for the early diagnosis of common maternal fetal abnormalities. This work has a few dark sides: (i) An imbalanced dataset presents challenges for training a deep learning model and can lead to the extraction of irrelevant information from deeper layers. In the future, the imbalance will be addressed by employing the generative algorithm and a residual attention module will be proposed to overcome the irrelevant information extraction. Our findings, based on the results, are as follows:

- Designed two novel CNN architectures: 3-Residual and 4-Residual-block architectures reduced the total learnable and complex, layered structures; however, this structure improved the learning of input dataset images and returned higher training accuracy.
- The 3-Residual model uses fewer parameters than ResNet 18 and ResNet 50, ensuring computational efficiency with competitive performance. The 4-Residual-block model adds hidden layers and reduces max-pooling for streamlined efficiency with fewer parameters.
- Enhanced the Generalized Normal Distribution Optimization (GNDO) algorithm for optimal feature selection, improving accuracy.
- The fusion of features improved accuracy, and with the application of an optimization algorithm, the overall framework was further enhanced in terms of accuracy, precision, and testing time.

## Data Availability

The original contributions presented in the study are included in the article/supplementary material, further inquiries can be directed to the corresponding authors.
